# Analysis of Rowing Force of the Water Strider Middle Leg by Direct Measurement Using a Bio-Appropriating Probe and by Indirect Measurement Using Image Analysis

**DOI:** 10.34133/cbsystems.0061

**Published:** 2023-11-17

**Authors:** Kaoru Uesugi, Hiroyuki Mayama, Keisuke Morishima

**Affiliations:** ^1^ Department of Mechanical Systems Engineering, Ibaraki University, 4-12-1 Nakanarusawa-cho, Hitachi, Ibaraki 316-8511, Japan.; ^2^ Department of Mechanical Engineering, Osaka University, 2-1 Yamada-oka, Suita, Osaka 565-0871, Japan.; ^3^ Global Center for Medical Engineering and Informatics, Osaka University, 2-1 Yamada-oka Suita, Osaka 565-0871, Japan.; ^4^ Department of Chemistry, Asahikawa Medical University, 2-1-1-1 Midorigaoka-Higashi, Asahikawa, Hokkaido 078-8510, Japan.

## Abstract

Rowing force of the middle leg of a water strider is one of the important factors affecting water repellency and applications in biomimetics, biomechanics, and biology. However, many previous studies have been based on estimated leg rowing force and lack some credibility. Therefore, we tried to measure leg rowing force directly by a force transducer. In this article, we report the rowing force of water striders obtained by direct and indirect measurements. In the direct measurement, water striders were set onto a sensor system and the rowing force of a middle leg of the set water striders was directly measured using a bio-appropriating probe (BAP), a kind of hook. In the indirect measurement, water striders were not fixed and the rowing force of locomoting water striders was evaluated by image analysis using a high-speed camera. As a result, we determined the rowing force by the direct measurement to be 955 μN, while the rowing force by the indirect measurement was 493 μN. We considered that the indirect measurement might lack some credibility because half the propellant energy was lost in the indirect force measurement due to various other factors.

## Introduction

Water striders make their habitat on water surfaces such as on ponds, brooks, and even the open ocean. Water striders can move on the water surfaces by rowing, that is, kicking the water surfaces using their legs. The leg rowing force of this insect species is an important factor from the viewpoints of water repellency and biomechanics.

**Fig. 1.**
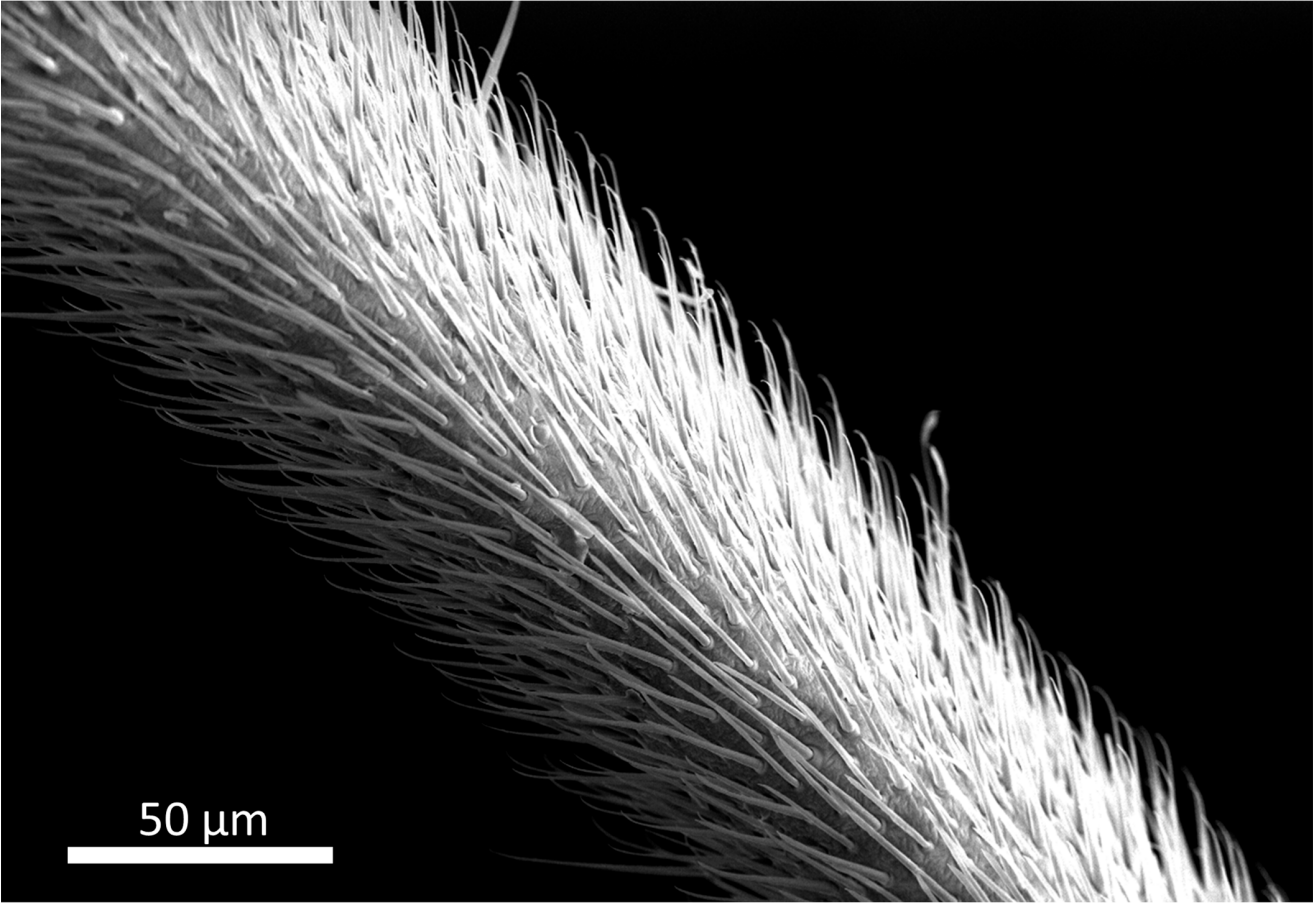
SEM image of microsetae of a water strider’s middle leg. Microsetae are roughly 50 μm in length and less than 3 μm in diameter.

First, we consider the viewpoint of water repellency. Water striders can float on water surfaces because of their super water-repellent legs that are covered by many microsetae (about 50 μm in length with a diameter less than about 3 μm) (Fig. 1). The microsetae form air layers between themselves, and the legs are not wetted by the water [1–5], which means that the microsetae result in the Cassie–Baxter state of wetting [1–3]. Actually, the microsetae structure has been mimicked by various techniques in the development of water-repellent materials [6–11]. If the rowing force exceeds the required force by too much, water can enter into the space between the microsetae and the super water repellency function of the legs will decrease [12,13] and fluid friction resistance of the legs will increase simultaneously. This is related to the reason why raindrops do not penetrate into the space of the surface structure of lotus leaves [14]. The microstructure of the water strider’s legs has been studied in developing semi-aquatic devices and robots [10,15–21]. If more details about the rowing force are known, understanding will be deepened.

Next, we consider the viewpoint of biomechanics. Because there are a number of physical phenomena at the air–water interface, Hu et al. [22] calculated middle leg rowing force from this biomechanical viewpoint. Additionally, they discussed the propellant mechanism between adult water striders and water strider larva. Adult water striders use capillary waves for propulsion. But water strider larvae cannot use the capillary waves because their body is too small (Denny’s paradox [23]). From their calculations, Hu et al. proposed the answer for this paradox: Water strider larvae transferred momentum to the water under them by using hemispherical vortices that were generated by middle leg strokes. Suter et al. [24] proposed the answer for this paradox in the earliest stage when people were trying to find an answer to the paradox. They showed that when the rowing speed at the tip of a fisher spider’s leg was slower than the capillary waves, drag resistance by dimples formed between the tips of the fisher spider’s rowing legs and the water surface was dominant. They mentioned that this theory can be appropriated to the locomotion of very small walking-on-water organisms like first-instar water striders. In order to discuss the trade-off relationship between flight and water locomotion in sexual conflicts, Goodwyn and Fujisaki [25] calculated middle leg rowing force by measuring propellant force. Water striders also show jumping motions on water surfaces. Biologically, researchers from 2 groups hypothesized that the leg lengths and leg stroke (stroke speed or stroke force) in the jump motion might be optimally adjusted for movement on water surfaces [26,27]. Proving this hypothesis might answer the question as to whether this adjustment was achieved by natural selection or by individual learning. In addition to this, it is known that the leg force (jump height) and morphology (length of the middle legs) were related by the Hox protein Ultrabithorax in evolutionary developmental biology [27]. Some robots mimicking water striders were also used to clarify the mechanisms of locomotion, which were explained above (simple locomotion and jumping locomotion) [22,28]. Information about the rowing force of water striders can also be used for development of semi-aquatic devices and robots. The propelling mechanisms of water striders have been intensively studied [4,22,29,30].

In order to understand the water strider locomotion, the force generated in the locomotion has been measured. The relationship between the whole leg rowing force applied on the body of a water strider and the rowing motion of its legs was discussed by direct and indirect force measurements [22,25,26,28,31,32]. The indirect force measurements estimated the value of leg rowing force [22,26,28,32]. The direct force measurements were carried out using a force transducer, while the indirect force measurements were carried out by calculating acceleration [22,32] or by measuring the shape of dimples in the water surface obtained from image analysis of the motions [26,28]. Propellant force of water striders was measured directly by a force transducer in 2 studies [25,31]. The propellant force was defined as the force applied to the water strider’s body, and it was the sum of the rowing forces and resistance forces of all legs. Then, these authors [25,31] calculated rowing force using the propellant force. If only the pure leg rowing force can be measured, more accurate and more sophisticated discussions would be possible.

There have been a lot of studies that directly measured leg force of various small animals, such as locusts [33,34], cockroaches [35–37], stick insects [38,39], drosophilae [40], and geckos [41,42]. Measuring leg force of small animals was mainly done by 3 methods. The first connected a leg to a force sensor by wires [34] or a stainless-steel pin [37]. The second embedded force sensors in part of the behavior field [33,38] or attached force sensors on the behavior field (e.g., force plate and force platform) [35,36,41,42]. The third used kicking of a force sensor probe by an animal, which was fixed onto a force sensor system [39,40].

However, it has been difficult to measure leg force of water striders as explained below. For example, when connecting a water strider’s leg to a force sensor, it was difficult to get natural movements of the water strider in the experimental setup. If an attempt was made to attach something on the strider body surface, the water striders reacted to it and moved unnaturally. A second reason was related to their habitat environments. If embedded force sensors or force plates were used, natural movements of animals occurred. In order to measure the water strider’s rowing force in a natural situation, however, the force sensor probe must be set close to the water surface because that was where the water strider moved, close to the water surface. Also, it was difficult to set the force sensors or force plates on the water surface since it was a liquid surface and unstable mechanically. Even if a probe was used to get kicking force of the water strider, it was difficult to measure the force accurately. When the water surface became wavy due to the rowing motion of the water strider, the sensor probe touched the water and was caught by the meniscus. Then, the force measurement was affected by the surface tension. Additionally, it was considered that the friction force and viscosity resistance between the middle leg of the insect and the water surface also affected force measurement. This was the main reason why accurate force measurements were difficult to make. Another was that water striders had microsetae on their body surface for water repellency and their body was small. These characteristics made it difficult to fix the body onto the force measurement system. Additionally, since the time scale of the locomotion was several milliseconds, a high-speed force measurement system was required. These conditions have made it difficult to measure force in natural movements.

**Fig. 2.**
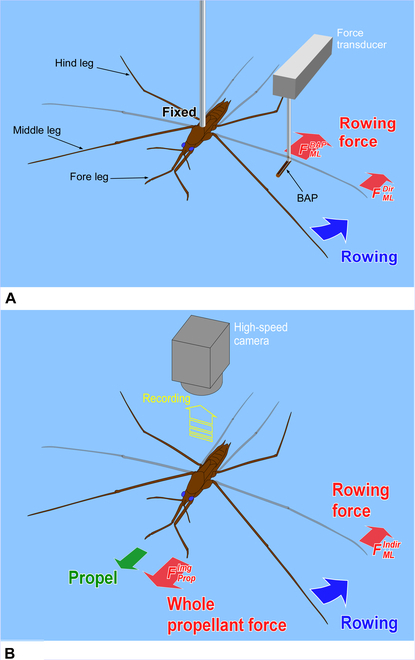
Schematic illustration of measurement of leg rowing force of water strider. (A) Direct force measurement using a force transducer. (B) Indirect force measurement using image analysis.

In order to resolve these difficulties, in this study, we proposed a new method and system that could measure a water strider’s rowing force directly. As shown in Fig. 2A, we prepared a new device, a bio-appropriating probe (BAP), and we improved the way by which it was fixed onto the water strider. As a result, the experimental problems outlined above were solved. Our direct force measurement could measure pure rowing force of the middle legs without effects from other factors. We estimated rowing force by image analysis to confirm the value of measured rowing force of the middle legs as an indirect measurement as shown in Fig. 2B. A few studies have compared results of the direct and indirect force measurements. Suter et al. [24] compared the locomotion motion of fisher spiders analyzed by image analysis and direct force measurement (using a force sensor). However, they only measured the drag force between the fixed leg (leg segment) and the water flow using the force sensor. They did not measure the rowing leg force of the living and naturally moving organism. Thus, by determining and comparing the rowing forces that were measured by the direct and indirect force measurements, we could discuss various properties of the water striders such as their water repellency, and we could also discuss applications to biomechanics and biology. Additionally, we analyzed the maximum force arrival time and the middle leg angular velocity of the direct and indirect force measurements.

## Materials and Methods

### Insects

We used adult female *Aquarius paludum paludum* (sixth instar), collected from a pond in Osaka University, Suita campus (Suita, Osaka). Conditions of samples were as follows. In the direct measurement, weights were about 39.4 mg (sample 1), 31.7 mg (sample 2), and 38.9 mg (sample 3) and numbers of trials were *n* = 3 (sample 1), *n* = 3 (sample 2), and *n* = 10 (sample 3). In the indirect measurement, we used different individuals and weights of samples were about 31.3 mg (sample 4), 37.3 mg (sample 5), and 36.5 mg (sample 6). Numbers of trials were *n* = 8 (sample 4), *n* = 13 (sample 5), and *n* = 15 (sample 6). The average of all weights of the insects that we used was 35.9 ± 3.5 mg (mean ± SD).

### Measurement system

Many studies have measured a small animal’s leg force directly, as detailed in the Introduction section. However, it is difficult to measure leg force of semi-aquatic insects such as water striders, because there are a lot of uncontrollable factors. Therefore, we newly constructed a leg force measurement system for water striders by referring to these conventional studies.

**Fig. 3.**
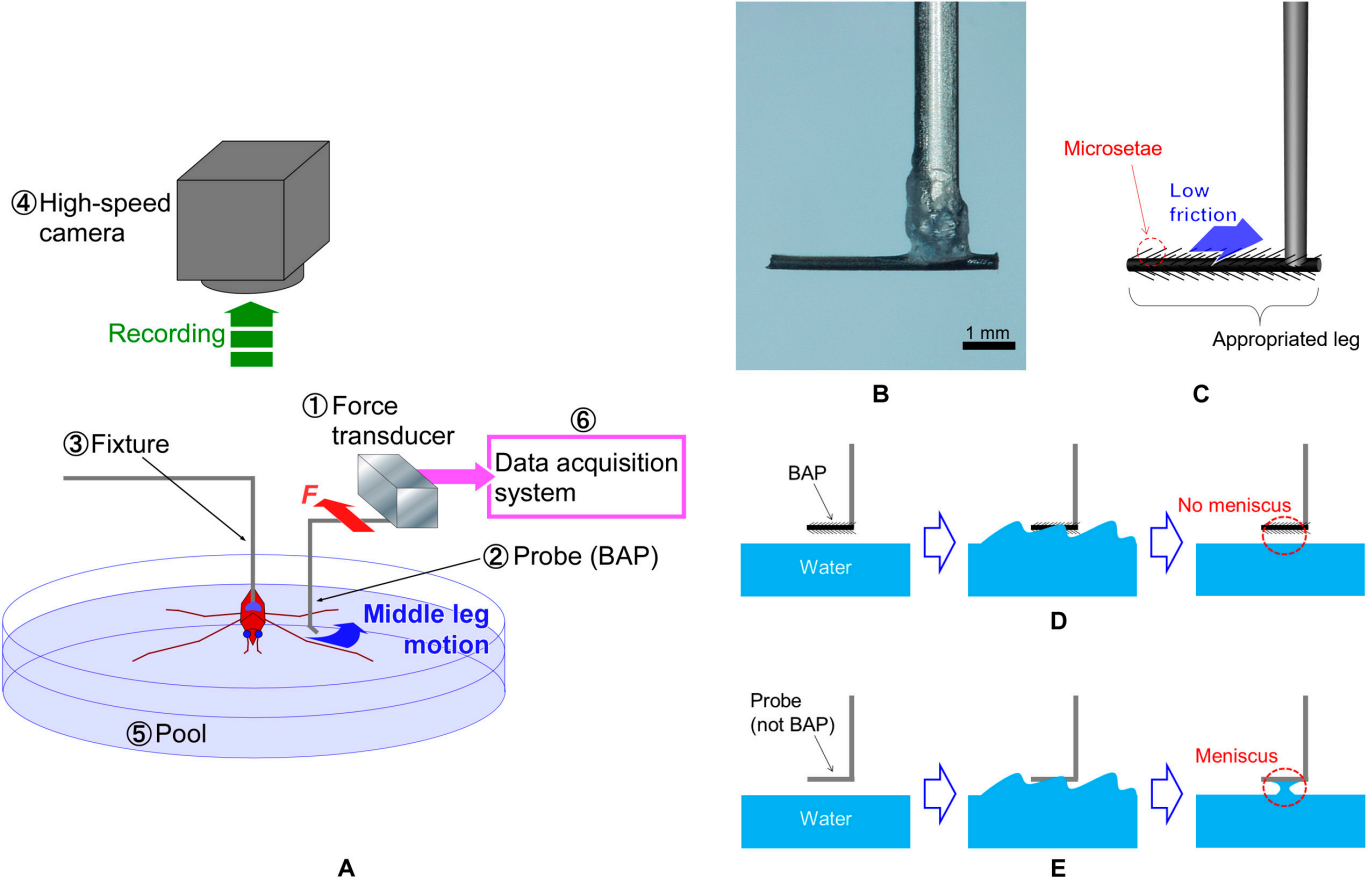
Schematic illustration of the force measurement system and the BAP to obtain rowing leg force of water strider. (A) The system consisted of 6 parts as follows: (1) a force transducer, (2) a BAP, (3) a fixture, (4) a high-speed CCD camera, (5) a pool, and (6) a data acquisition system. (B) Photo of the BAP. (C) Schematic illustration of the BAP. The base of the L-shape appropriated part of the femur of the water strider’s leg. (D) The BAP can repel water, and it is not caught by the meniscus when the water surface is close to the BAP. (E) If the BAP is not used, the probe is caught by the meniscus.

Figure 3A shows a schematic illustration of the rowing force measurement system. The measurement system consisted of 6 components: (a) a force transducer (FRS-711, TECH ALPHA, Tokyo) (range, 0.00 to 33.6 mN; resolution, 3.4 μN) to measure water strider leg force, (b) a bio-appropriated probe (BAP) to catch a water strider’s leg by hooking it and to transfer the leg force to the force transducer, (c) a fixture to attach the water strider’s body to the force measurement system, (d) a high-speed video camera (IDP, Photron, Tokyo) to observe propulsion motion of the water strider, (e) a water pool consisting of a petri dish (diameter: 15 mm) to imitate a semi-aquatic environment, and (f) a data acquisition system (PCI-6036A LabVIEW, National Instruments Co., Texas) to store force signals of a water strider’s leg force in a personal computer. Since the time scale of the leg motion of one rowing stroke was several hundred milliseconds, the frame rate of the high-speed camera was 1,000 fps. As the water strider and the probe were close to the water pool surface mimicking the water strider’s habitat, we were able to reproduce the natural condition for water striders on the water surface. The BAP was hooked close to the middle leg part between the femur and tibia (Fig. S1 shows regions of the water strider). It was too difficult to set the BAP close to the tip of the middle leg (around the tarsus) because this leg tip was in contact with the water surface, and the BAP became immersed in the water and the force measurement was affected by the viscosity resistance.

### The bio-appropriating probe

Figure 3B shows a side view of the sensor probe, the BAP. It was L-shaped to allow hooking of a leg. In the experiments, the BAP was positioned at 1 to 2 mm away from the water surface because the propulsion movement of the water strider on this surface was observed at the same distance. Part of the femur of the water strider’s leg was used as the base of the L-shaped BAP as shown in Fig. 3C. The water strider’s leg, which has super water repellency, was used to prevent a meniscus from forming between the BAP and water surface as shown in Fig. 3D. When we used a probe that did not have sufficient water repellency, the probe touched the wavy water surface that was produced by the water strider rowing its legs. A meniscus was formed between the probe and water surface, and accurate force measurement could not be carried out (Fig. 3E).

### Direct measurement of water strider leg force using the BAP

**Fig. 4.**
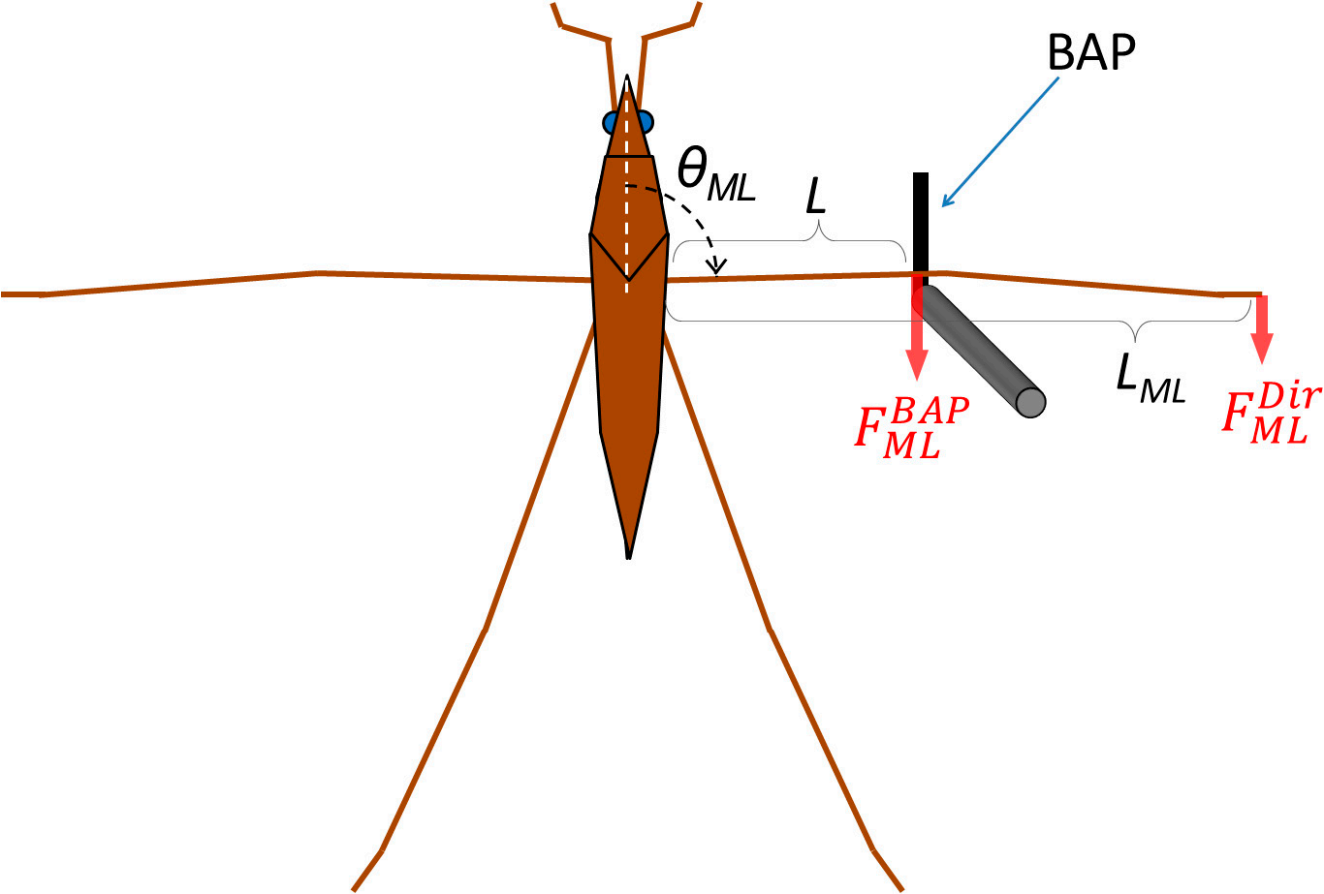
Model for calculation of rowing force of the middle leg tip. $F_{\mathrm{ML}}^{\mathrm{Dir}}$ is the calculated rowing leg force, and $F_{\mathrm{ML}}^{\mathrm{BAP}}$ is the measured middle leg rowing force obtained with the BAP. *L_ML_* is the overall length of the middle leg, and *L* is the middle leg length between the femur and tibia. *θ_ML_* is the angle of the middle leg.

The procedure for the direct measurement of rowing leg force is as follows. First, a water strider’s body was attached to the fixture of the sensor system (Supplementary Method and Fig. S2). Then, the BAP was positioned at 1 to 2 mm height from the water surface. In order to carry out force measurements under uniform conditions, the position of the BAP was adjusted at the point where the angle between the middle leg and body became about 90° when the middle leg was hooked by the BAP (in Fig. 4, *θ_ML_* = 90°). When the water strider rowed with its legs, the BAP hooked onto a joint between the femur and tibia of a middle leg. Thus, the BAP measurement of rowing force between this femur and tibia of the middle leg ($F_{\mathrm{ML}}^{\mathrm{BAP}}$) was carried out along the horizontal direction. The previous studies that are compared with our results in the “Discussion” section discussed horizontal direction force [25,31,32]. Therefore, in this study, we measured the horizontal direction force.

Because of the principle of the force transducer, we restricted (fixed) the movement of the leg of the water strider during the force measurement. In the direct force measurement, the body of the water strider was fixed mechanically to the measurement system, and when the water strider rowed its legs, the leg used for the measurement was hooked by the probe. The downward direction and backward direction motion of the middle leg hooked by the BAP were constrained during the force measurement. We considered that the change of the vertical direction distance between the body and the force-measuring point of the middle leg was small during the force measurement because the vertical movement of the body was also constrained. Therefore, we thought that the fixing of the body did not affect the rowing force substantially because the leg rowing force was measured by the force transducer without leg motion (isometric force measurement). Therefore, leg force was measured with a small energy loss.

When the motion of rowing was not natural, the measured force data were rejected (e.g., when the middle leg joint was largely bent while held by the probe, or the middle leg was largely lifted up, or the legs were thrashing about). Additionally, in the observations, in order to obtain the rowing force for straight propelling naturally, we confirmed that both left and right middle legs were equally rowed. As samples, 3 adult female water striders were used (samples 1 to 3). Details of the number of measurements for each individual are given in Table S1. $F_{\mathrm{ML}}^{\mathrm{BAP}}$ was expressed as the mean ± SD.

### Observation of motion of water strider using a high-speed camera for indirect measurement of water strider leg force

We investigated a correlation between the motion and the propellant force by image processing to understand the relationship between the propellant force and the rowing force. Figure 2B shows the setup for indirect rowing force measurement. A white mark was painted on the water strider pronotum using acryl gouache, and the insect was put into an aquarium (W, 35 cm; L, 50 cm; H, 30 cm) with water to a depth of about 3 cm. Then, a movie of its high-speed movement by rowing of the middle legs was captured by a high-speed charge-coupled device (CCD) camera (Photron IDE, Photron Ltd., Tokyo) (lens for OLYMPUS OM: 28mm f2.5 MC SOLIGOR WIDE-AUTO, A.I.C. Phototechnik GmbH, Land Baden-Württemberg) (lens adapter: C-MOUNT ADAPTER for OLYMPUS OM, Kenko, Tokyo) at the recording speed of 1,000 fps. The white mark was followed by image analysis software (Dipp-Motion V, DITECT Co. Ltd., Tokyo), and its velocity was derived. Then, its maximum acceleration rate was calculated from the change of velocity. In this experiment, the acceleration of 3 adult female water striders was obtained (samples 4 to 6). Details of the number of measurements for each individual are given in Table S1.

## Analysis

### Derivation of rowing force of a middle leg by direct force measurement

We determined the middle leg rowing force that was measured with the BAP as $F_{\mathrm{ML}}^{\mathrm{BAP}}$. Figure 4 shows the geometry and definitions of characteristic sizes of the water strider to calculate the rowing force that acts on the tip of a middle leg. The $F_{\mathrm{ML}}^{\mathrm{BAP}}$ determined by the BAP is the force of a middle leg in the part between its femur and tibia; therefore, it is not the force generated on the tip (the point where the force acts onto the water surface). The symbols are summarized in Table 1. To evaluate the force at the tip, we determined the directly measured maximum rowing force that was generated on the tip of the middle leg (ML) as $F_{\mathrm{ML}}^{\mathrm{Dir}}$. $F_{\mathrm{ML}}^{\mathrm{Dir}}$ was defined by calculation of the moment using Eq. 1.

$$F_{\mathrm{ML}}^{\mathrm{Dir}}=\left(L/L_{\mathrm{ML}}\right)F_{\mathrm{ML}}^{\mathrm{BAP}}$$ (1)

**Table 1.** Summary of types of forces, symbols, and equations

Method	Direct force measurement (force transducer)		Indirect force measurement (image analysis)	
Symbol	$F_{\mathrm{ML}}^{\mathrm{BAP}}$	$F_{\mathrm{ML}}^{\mathrm{Dir}}$	$F_{\text{Prop}}^{\mathrm{Img}}$	$F_{\mathrm{ML}}^{\text{Indir}}$
Applied force	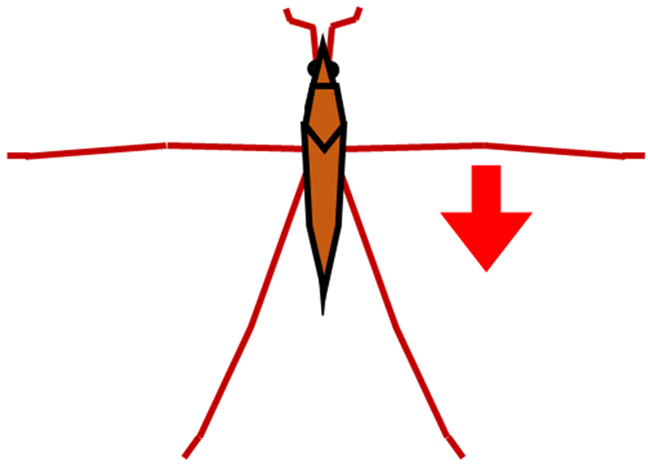	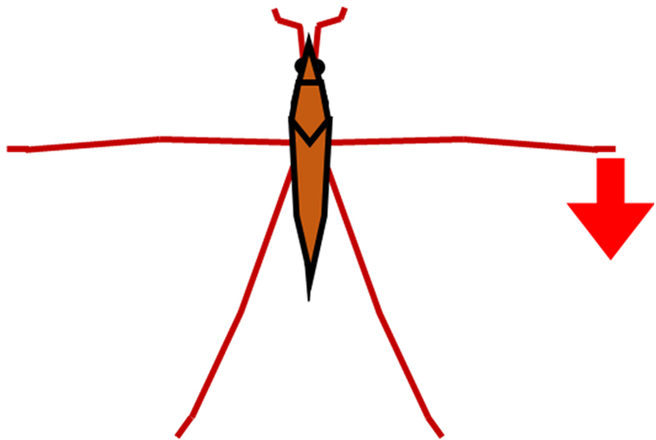	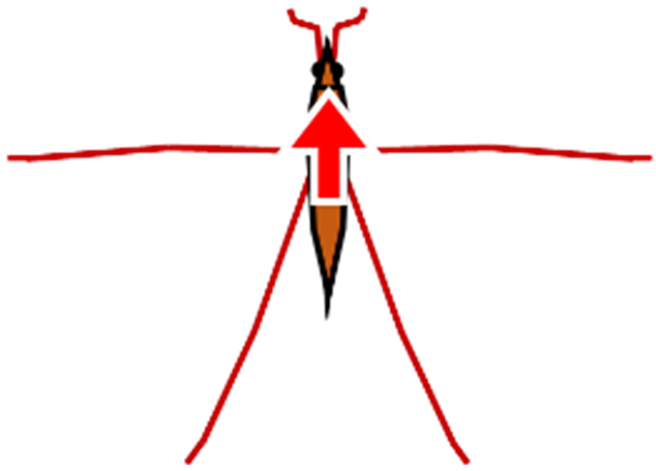	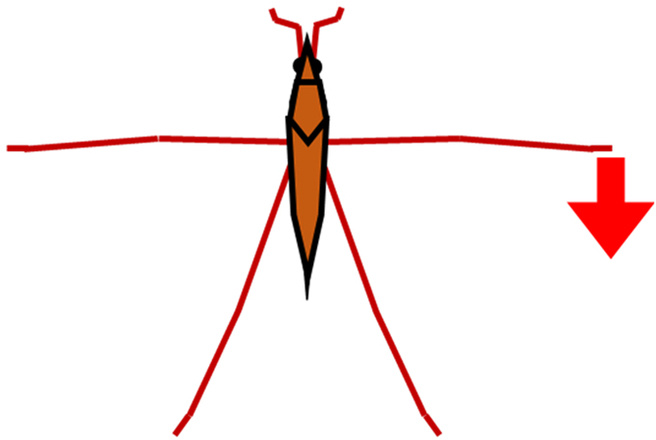
Details	Measured rowing force between femur and tibia of middle leg with the BAP	Calculated rowing force of middle leg calculated from $F_{\mathrm{ML}}^{\mathrm{BAP}}$	Whole-body propellant force derived from the image analysis	Calculated rowing force of middle leg calculated from $F_{\text{Prop}}^{\mathrm{Img}}$
Equation	-	Eq. 1	Eq. 2	Eq. 3

Here, *L_ML_* is the length of a stretched middle leg and *L* is the length between the femur and tibia of the middle leg as shown in Fig. 4. $F_{\mathrm{ML}}^{\mathrm{Dir}}$ was expressed as the mean ± SD. In this analysis, we modeled the leg as a single beam with a constant moment (see Supplementary Analysis including Fig. S3).

### Derivation of rowing force of a middle leg by indirect force measurement

In order to derive middle leg rowing force by image analysis, we determined the whole propellant force $F_{\text{Prop}}^{\mathrm{Img}}$, which was the sum of the forces generated with all legs. $F_{\text{Prop}}^{\mathrm{Img}}$ was calculated by Eq. 2. The symbols are summarized in Table 1.

$$F_{\text{Prop}}^{\mathrm{Img}}={\mathrm{ma}}_{\text{Prop}}^{\mathrm{Img}}$$ (2)

Here, *m* is the weight of a water strider and $a_{\text{Prop}}^{\mathrm{Img}}$ is the maximum acceleration for propellant that was obtained by the image analysis. Then, the middle leg rowing force was derived by Eq. 3.

$$2F_{\mathrm{ML}}^{\text{Indir}}=0.7F_{\text{Prop}}^{\mathrm{Img}}$$ (3)

$F_{\mathrm{ML}}^{\text{Indir}}$ is the maximum rowing force of a middle leg, and it is evaluated from $F_{\text{Prop}}^{\mathrm{Img}}$ in Eq. 2. The factor of 2 in the left side of Eq. 3 means that both middle legs are rowed simultaneously when the water strider is propelling itself. On the other hand, the factor of 0.7 is based on the experimental findings that 70% of the propellant force is generated by the middle legs [25,31]. Because we observed horizontal movement of water striders, $F_{\mathrm{ML}}^{\text{Indir}}$ is also along the horizontal direction like $F_{\mathrm{ML}}^{\mathrm{Dir}}$ $\left(F_{\mathrm{ML}}^{\mathrm{BAP}}\right)$. $F_{\mathrm{ML}}^{\text{Indir}}$ was expressed as the mean ± SD.

### Arrival time of maximum force

**Fig. 5.**
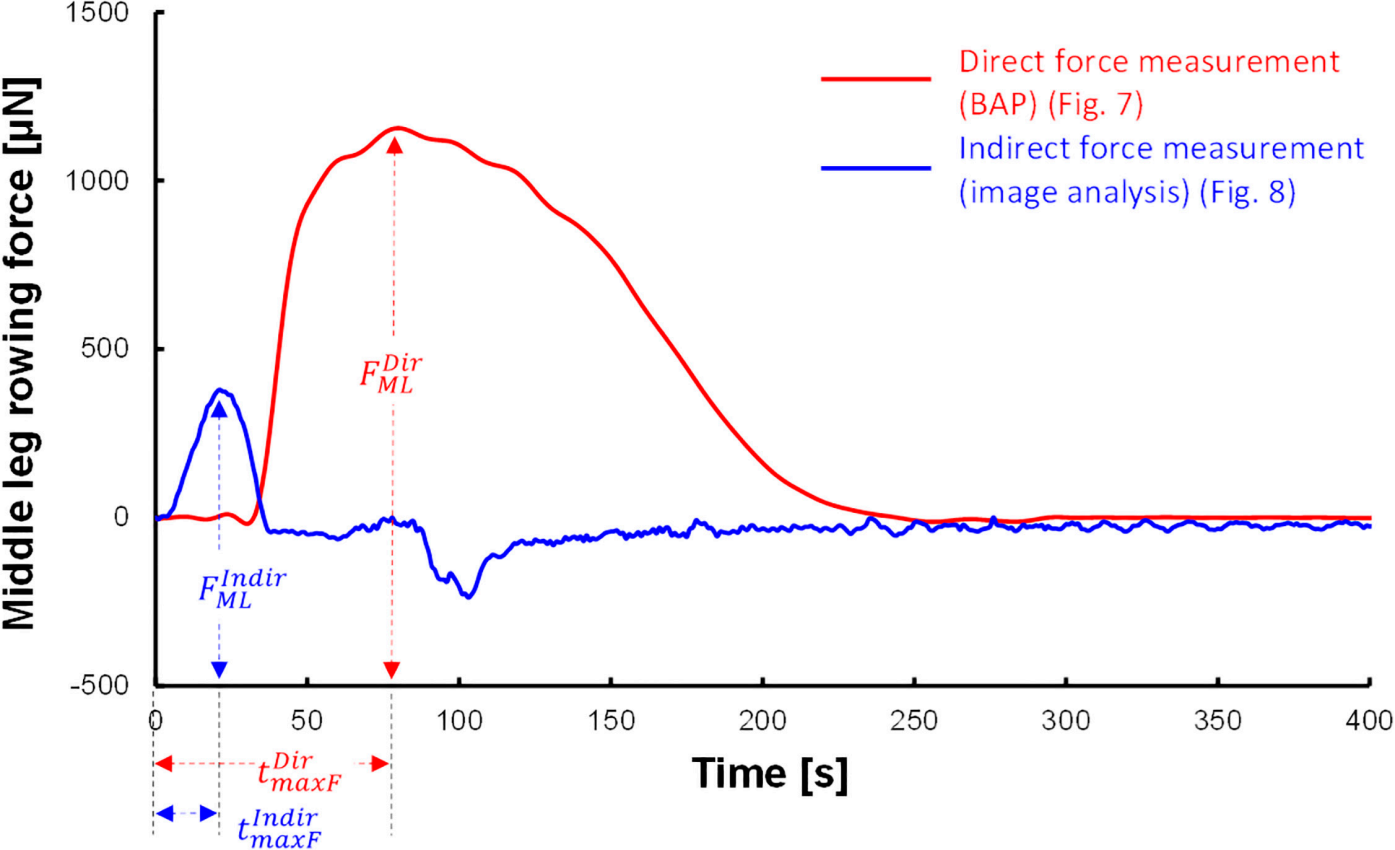
Schematic diagram showing individual leg rowing forces and maximum force arrival times obtained in this research (example of obtained data). The red curve is the directly measured leg rowing force (Fig. 6), while the blue curve is the indirectly measured leg rowing force (Fig. 7). $F_{\mathrm{ML}}^{\mathrm{Dir}}$ and $F_{\mathrm{ML}}^{\text{Indir}}$ are defined by the maximum rowing force of the direct and indirect force measurement. Their respective maximum force arrival times ($t_{\text{maxF}}^{\mathrm{Dir}}$, $t_{\text{maxF}}^{\text{Indir}}$) are the times taken to reach maximum leg rowing force from the starting time of leg rowing.

We obtained arrival times of maximum forces to allow comparison of the rowing motion and the force generation observed by the direct force measurement using the BAP and indirect force measurement using image analysis. The arrival times of maximum force are defined as $t_{\text{maxF}}^{\mathrm{Dir}}$ and $t_{\text{maxF}}^{\text{Indir}}$ for the direct and indirect force measurements (Fig. 5), and they are the times needed to generate the maximum force. $t_{\text{maxF}}^{\mathrm{Dir}}$ and $t_{\text{maxF}}^{\text{Indir}}$ were expressed as mean ± SD.

### Angular velocity of leg rowing motion

Angular velocity was evaluated to understand the relationship between rowing motion of the middle leg and generation of force. The angular velocities in the direct and indirect observations were roughly evaluated by Eq. 4.

$$\omega_{\mathrm{ML}}^{i}=\frac{∆\theta^{i}}{∆t^{i}}\approx \frac{90{}^{\circ}-\theta^{i}\left(t=0\right)}{∆t^{i}}$$ (4)

Here, *i* = *Dir* and *Indir*; $\omega_{\mathrm{ML}}^{\mathrm{Dir}}$ and $\omega_{\mathrm{ML}}^{\text{Indir}}$ are the angular velocities of leg rowing motion for direct and indirect force measurements, respectively; $\theta_{\mathrm{ML}}^{i}$ is the angle between the middle leg and body as shown in Fig. 4; ∆*t^i^* is the arrival times to reach 90^°^ from the initial angle; and *θ^i^*(*t* = 0) is the initial angle at *t* = 0. $\omega_{\mathrm{ML}}^{\mathrm{Dir}}$ and $\omega_{\mathrm{ML}}^{\text{Indir}}$ were expressed as the mean ± SD.

## Results

**Fig. 6.**
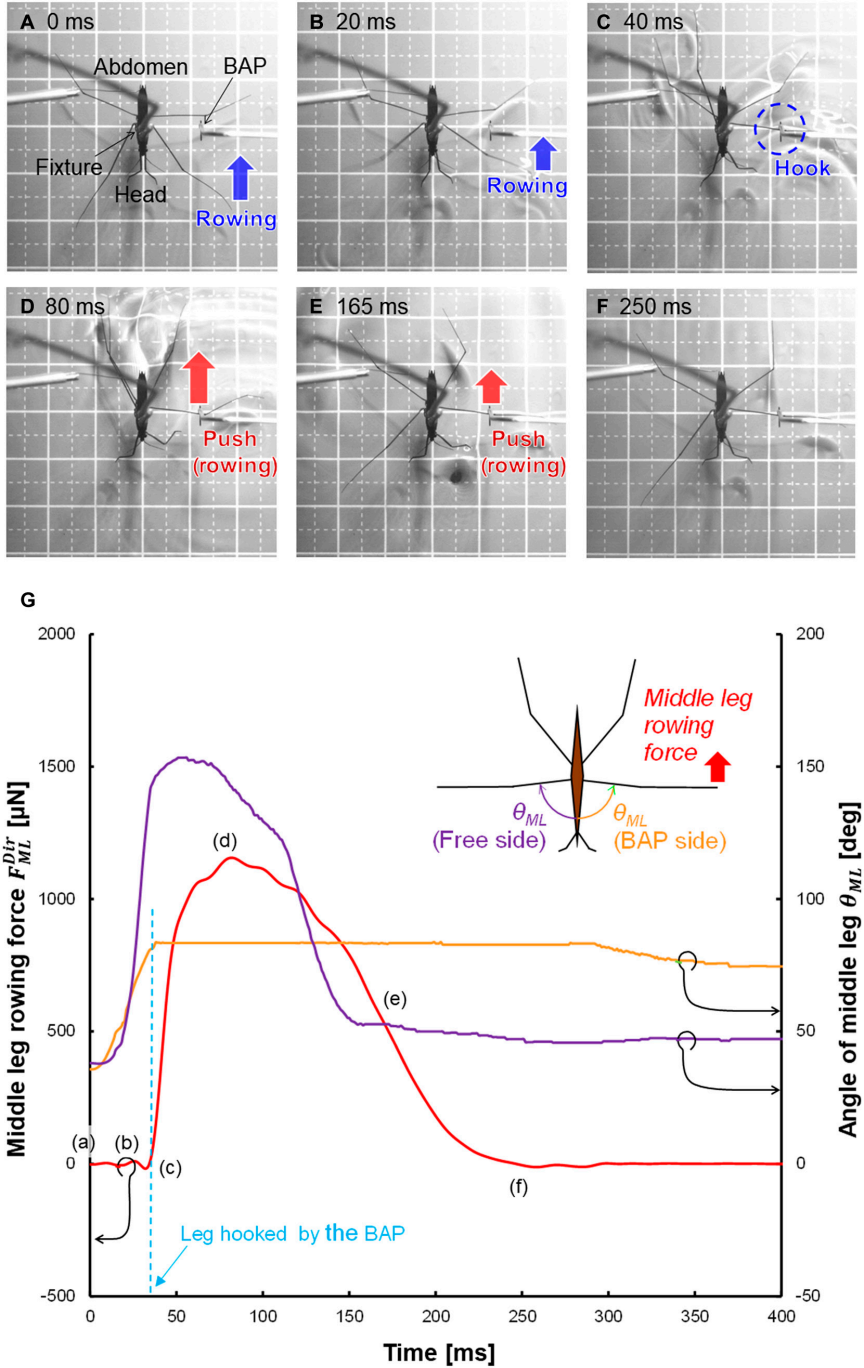
Detailed state of the direct force measurement. (A to F) Photos of a water strider. The bold squares in each photo are 10 mm on a side. (G) Results of the direct measurement of the rowing force of a middle leg measured by the BAP $F_{\mathrm{ML}}^{\mathrm{Dir}}$ (red line) and for 2 middle leg angles *θ_ML_* (orange and purple lines). The letters identifying middle leg rowing force curves correspond to the water strider position in the photos at the time of measurement. The same data are shown in Fig. 5, where they are compared with the propellant force determined by indirect measurements based on image analysis as explained in the text.

**Fig. 7.**
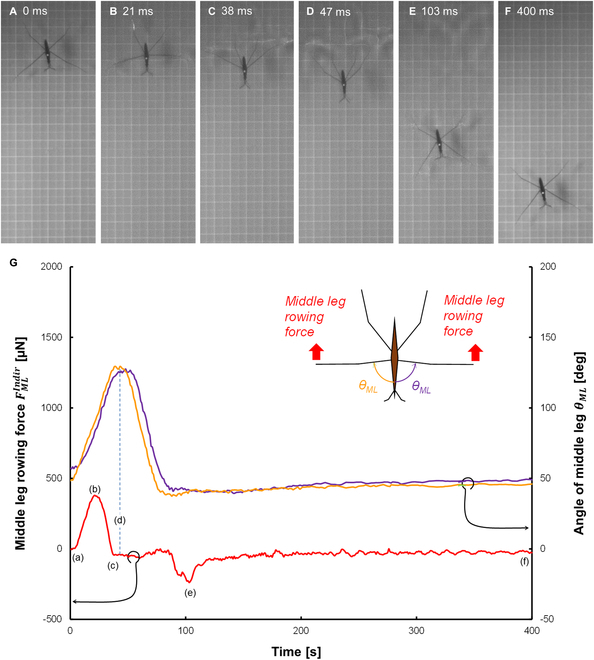
Detailed state of the indirect force measurement. (A to F) Photos for the indirect measurement of rowing force of a middle leg. The bold squares in each photo are 10 mm on a side. (G) Results of rowing force of a middle leg $F_{\mathrm{ML}}^{\mathrm{Indir}}$ (red line) and 2 middle leg angles *θ_ML_* (orange and purple lines) determined by image analysis. The same data are also shown in Fig.5.

Figure 5 overlays the results of the middle leg rowing forces obtained by the direct and indirect measurements for comparison. Clearly, the maximum force of $F_{\mathrm{ML}}^{\mathrm{Dir}}$ was larger than that of $F_{\mathrm{ML}}^{\text{Indir}}$, but $t_{\text{maxF}}^{\mathrm{Dir}}$ was later than $t_{\text{maxF}}^{\text{Indir}}$. Here, we show these results in Figs. 6 and 7 independently and present the details in the “Discussion” section.

### Direct measurement of water strider leg force measured by BAP

**Fig. 8.**
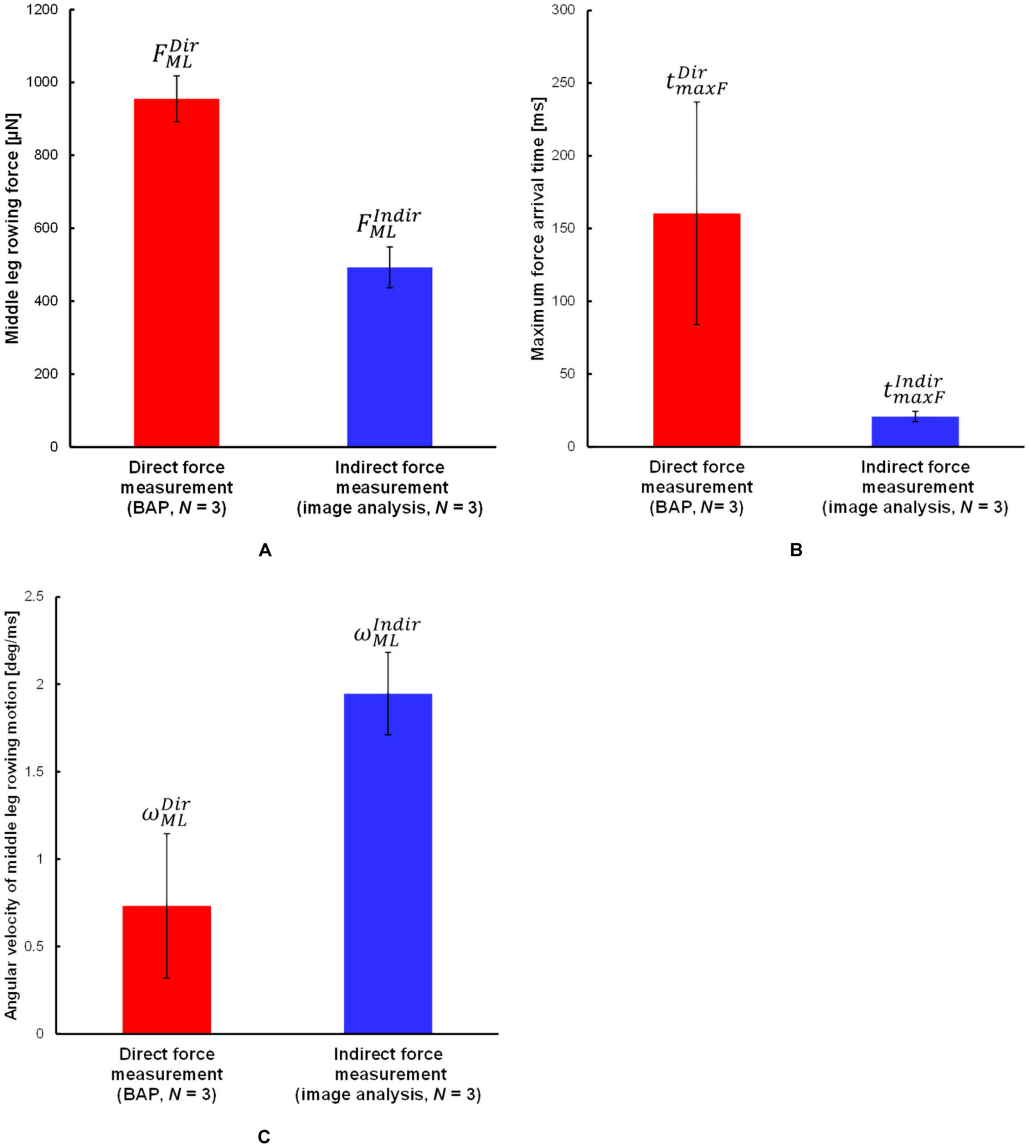
Data obtained by the direct force measurement with the BAP (red columns) and indirect force measurement measured by image analysis (blue columns). (A) Directly measured rowing force of a middle leg ($F_{ML}^{\mathrm{Dir}}$, 955 ± 63 μN) and indirectly measured rowing force ($F_{ML}^{\text{Indir}}$, 493 ± 56 μN). (B) Maximum force arrival time of a middle leg of the direct measurement ($t_{\text{maxF}}^{\mathrm{Dir}}$, 160.4 ± 76.4 ms) and that of the indirect measurement ($t_{\text{maxF}}^{\text{Indir}}$, 20.8 ± 3.6 ms). (C) Angular velocity of lowing motion of a middle leg of the direct measurement ($\omega_{\mathrm{ML}}^{\mathrm{Dir}}$, 0.73 ± 0.41 deg/ms) and that of the indirect measurement ($\omega_{\mathrm{ML}}^{\text{Indir}}$, 1.95 ± 0.24 deg/ms). Data were expressed as mean ± SD. The number of individuals used in each measurement was 3. (Details of the number of measurements, mean, and SD for each individual are given in Table S1.)

The BAP had high water repellency because part of the femur of the water strider leg was used for the base of the L-shape of the BAP. Therefore, the BAP did not contact the water surface with its meniscus and the force could be measured accurately. The BAP could measure $F_{\mathrm{ML}}^{\mathrm{BAP}}$ for samples 1 to 3 without being effected by the meniscus (Movie S1). The maximum value of $F_{\mathrm{ML}}^{\mathrm{BAP}}$ was determined to be 2,172 ± 143 μN (all values are shown by the mean ± SD) (Table 3; more details about the values are shown in Table S1). Figure 6 and Fig. S4A show typical experimental results of the sample 3 water strider. Figure 6A to F shows photos taken during the experiment, and Fig. 6G shows the time courses of $F_{\mathrm{ML}}^{\mathrm{Dir}}$ and $\theta_{\mathrm{ML}}$ values of the middle leg hooked by the BAP and the free leg. The times when the middle leg movements started were almost the same. $F_{\mathrm{ML}}^{\mathrm{Dir}}$ $\left(F_{\mathrm{ML}}^{\mathrm{BAP}}\right)$ was detected after hooking the middle leg with the BAP (Fig. 6C), and the start time of increasing leg rowing force and the stop time of rowing motion (increment of the angle of the BAP side middle leg) corresponded. The maximum value of $F_{\mathrm{ML}}^{\mathrm{Dir}}$ was evaluated by Eq. 1 as 955 ± 63 μN (red column, Fig. 8A). When water striders rowed their middle legs, contact length between the middle leg and water surface was reported to be about 11.0 ± 1.1 mm [12]. Therefore, rowing force per unit length was about 87 μN/mm. $t_{\text{maxF}}^{\mathrm{Dir}}$ was 160 ± 76 ms (red column, Fig. 8B) and $\omega_{\mathrm{ML}}^{\mathrm{Dir}}$ was 0.73 ± 0.41 deg/ms (red column, Fig. 8C). The obtained values are summarized in Tables 1 and 3 (more details about the values are shown in Table S1).

### Indirect measurement of water strider leg force by image processing

Figure 7A to F shows photos of the water striders, and Fig. 7G plots typical experimental results of $F_{\mathrm{ML}}^{\text{Indir}}$ and $\theta_{\mathrm{ML}}$. The photos showed that the water strider started rowing both middle legs almost at the same time, and it was propelled straightly in the forward direction. In Fig. 7G, $F_{\mathrm{ML}}^{\text{Indir}}$ was generated with the increase in $\theta_{\mathrm{ML}}$. Negative force was observed around *t* = 100 ms (Fig. 7E), and it denoted a deceleration of propelling. The average of the maximum speed $v_{\max}^{\text{Indir}}$ by one rowing was 864 ± 153 mm/s. The maximum value of $a_{\text{Prop}}^{\text{Img}}$ was about 40.3 ± 3.7 m/s^2^. Thus, the maximum value of $F_{\text{Prop}}^{\mathrm{Img}}$ (1,408 ± 161 μN) was derived by the motion equation (Eq. 2). By inserting $F_{\text{Prop}}^{\mathrm{Img}}$ in Eq. 3, the maximum value of $F_{\mathrm{ML}}^{\text{Indir}}$ was derived and the average calculated leg force was about 494 ± 56 μN (blue column, Fig. 8A). The rowing force per unit length was about 45 μN/mm (contact length: about 11.0 mm). $t_{\text{maxF}}^{\text{Indir}}$ was 21 ± 4 ms (blue column, Fig. 8B), and $\omega_{\mathrm{ML}}^{\text{Indir}}$ was 1.95 ± 0.24 deg/ms (blue column, Fig. 8C). In particular, comparing Figs. 6G and 7G, we found a difference in the relationship between the rowing manner of middle legs and the generation of rowing force. In Fig. 7G, the rowing motion of the middle legs generated rowing force continuously. In contrast, in Fig. 6G, there was a time lag between the detection of force and the change in $\theta_{\mathrm{ML}}$. This was due to the time lag between the hooking and the detection. Furthermore, the change in of the indirect force measurement $\theta_{\mathrm{ML}}$ was seen to be half of a sine wave, but the change in of the direct force measurement $\theta_{\mathrm{ML}}$ was asymmetric with a long tail. The latter behavior was due to the water striders being forced to continue to push the BAP. The obtained values were summarized in Tables 2 (more details about the values are shown in Table S1).

## Discussion

The maximum rowing forces, the arrival time of maximum force, and the angular velocities obtained by the direct and indirect measurements are compared in Fig. 8. Figure 8A shows that the water striders generated a larger rowing force than that in the indirect measurement. Figure 8B indicates that the water striders took some time to generate the maximum rowing force in the direct measurement. In contrast, angular velocity in the indirect measurement was faster than that in the direct measurement (Fig. 8C). Based on these results, the nonfixed water strider generated a smaller rowing force, but the rowing motion of the nonfixed water strider was momentary.

The BAP had high water repellency because part of the femur of the water strider leg was used for the base of the L-shape of the BAP. Therefore, the BAP did not contact the water surface with its meniscus and the force could be measured accurately. We could measure the middle leg force directly without various interferences like the effect of the meniscus. Because the length of the whole middle leg *L_ML_* was over twice the length between the body and the probe contact area (between the femur and tibia) *L*, $F_{\mathrm{ML}}^{\mathrm{Dir}}$ was about 955 ± 63 μN (red column; Fig. 8A), which was less than half the measured force $F_{\mathrm{ML}}^{\mathrm{BAP}}$ (2,172 ± 143 μN).

We calculated leg force $F_{\mathrm{ML}}^{\text{Indir}}$ by image analysis (blue column; Fig. 8A) for comparison with measured leg force $F_{\mathrm{ML}}^{\mathrm{Dir}}$. $F_{\mathrm{ML}}^{\text{Indir}}$ was about half $F_{\mathrm{ML}}^{\mathrm{Dir}}$. $F_{\mathrm{ML}}^{\mathrm{Dir}}$ was larger than 100% of the middle leg force (704 μN), which was derived from $F_{\mathrm{ML}}^{\text{Indir}}$ [the calculated middle leg rowing force was considered to be 70% of the whole propellant force (see the “Analysis” section)]. We thought that the reason why $F_{\mathrm{ML}}^{\text{Indir}}$ was about half of $F_{\mathrm{ML}}^{\mathrm{Dir}}$ was due to part of the energy for propelling generated by a leg being lost. When a water strider propels itself across a water surface, there are resistance factors such as friction force between the legs and water surface, air resistance, strain of the legs, and deformation of the water surface (viscoelasticity). When the water strider is propelling itself, it jumps up from the water surface and perpendicular force is reduced. Therefore, friction force is reduced and force transfer efficiency is also reduced (legs may swing without kicking the water surface). Additionally, because the force application point (capillary waves of the water surface made by the leg rowing motion) generally moved in the opposite direction to the propelled direction, the acting force and the acting time of the force were decreased. This indicated one possible explanation was that transmission of rowing leg force was cancelled by movement of the force application point (Fig. S5). Because the relative speed between the force application point and the body increased, the rowing speed of the middle leg tip could not catch up with the relative speed of the force application point. Then, the propellant force of the middle leg could not be transmitted to the water surface. Therefore, all leg rowing force might not be transformed to propellant force.

The effect of movement of the application point was confirmed by the relationship between the maximum force arrival time and the angular velocity of leg rowing motion. Despite $\omega_{\mathrm{ML}}^{\text{Indir}}$ (blue column, Fig. 8C; 1.95 ± 0.24 deg/ms) being only 2.7 times faster than $\omega_{\mathrm{ML}}^{\mathrm{Dir}}$ (red column, Fig. 8C; 0.73 ± 0.41 deg/ms), $t_{\text{maxF}}^{\text{Indir}}$ (blue column, Fig. 8B; 21 ± 4 ms) was 7.6 times shorter than $t_{\text{maxF}}^{\mathrm{Dir}}$ (red column, Fig. 8B; 160 ± 76 ms). We thought that because the point of force application moved, transmission of rowing force to this point was finished earlier.

We considered the reasons why $\omega_{\mathrm{ML}}^{\mathrm{Dir}}$ and $\omega_{\mathrm{ML}}^{\text{Indir}}$ were different. The rowing speed obtained by the direct force measurement could be decreased by the effect of friction force between the middle leg and water surface caused by the insect’s body. Because the fixture was attached on the water strider in a vertical direction, the downward force loaded on the body was more than in a natural situation and this caused deeper sinking of the legs with a larger meniscus being formed. Then, the friction force between the leg and the water surface would become larger than in the natural situation [43]. However, the friction force did not have a substantial effect on our direct force measurement because one middle leg that was rowing was held by the BAP and its movement was stopped so no friction force was being generated during the measurement. In the direct leg force measurement, we considered the model (Eq. 1) as rigid. There was a possibility that the deformation (bending) of legs caused energy consumption that in turn might cause the decrease of leg rowing force in the indirect leg force measurement. The energy consumption might be caused by deformation of the legs. Leg deformation when legs are continuously bending might consume energy, meaning that energy for propelling would be used for leg deformation and the force transmission efficiency from the legs to the water surface decreased. However, we confirmed from the movie taken by the high-speed camera in the indirect force measurement that deformation of the legs seemed to stop at the instant when *θ_ML_* = 90° was reached by the balance of the deformation and restoration or by keeping the degree of deformation. Therefore, we considered that the energy consumption due to the deformation of the leg of the indirect force measurement was small.

There are a few studies that have dealt with leg rowing force directly and indirectly. By comparing $F_{\mathrm{ML}}^{\mathrm{Dir}}$ and $F_{\mathrm{ML}}^{\text{Indir}}$, we could recognize that the middle leg rowing force that was measured by the direct force measurement was larger than that of the indirect force measurement. This meant that the rowing force that was generated from the middle leg was actually different from the rowing force that was calculated by image analysis.

Goodwyn and co-workers [25,31] presented 2 studies about direct measurement of propellant force of water striders (identified in their paper as *A. paludum*) and calculation of rowing force of a middle leg. In the first report, they used propellant force to calculate middle leg rowing force and obtained a value of approximately 1.36 to 1.71 mN (1,360 to 1,710 μN) [25] (Table 3). This value was larger than the rowing force $F_{\mathrm{ML}}^{\mathrm{Dir}}$ that we obtained in the present study. This could be accounted for by the difference in measurement methods. The measurement system of Goodwyn and Fujisaki [25] pushed downward more than in the natural situation, and this caused increased friction force between the water surface and legs to become larger than in the natural situation [43]. Therefore, the calculated rowing force of Goodwin and Fujisaki could become larger. In our measurement, the middle leg for which force was measured directly was held by the BAP and the leg did not sink deeply into the water. Additionally, because leg movement was stopped, friction force was not generated between measurements. Thus, the effect of this friction force was extremely small.

**Table 2.** Summary of obtained mechanical properties of propulsion

Mechanical properties		Symbol	Value (mean ± SD)	Figure
Direct measurement by BAP (*N* = 3)	Measured rowing force of a middle leg with the BAP (maximum value) (μN)	$F_{\mathrm{ML}}^{\mathrm{BAP}}$	2,172 ± 143	-
	Rowing force of a middle leg calculated from $F_{\mathrm{ML}}^{\mathrm{BAP}}$ (maximum value) (μN)	$F_{\mathrm{ML}}^{\mathrm{Dir}}$	955 ± 63	Fig. 8A
	Maximum force arrival time (ms)	$t_{\text{maxF}}^{\mathrm{Dir}}$	160 ± 76	Fig. 8B
	Angular velocity of leg rowing motion (deg/ms)	$\omega_{\mathrm{ML}}^{\mathrm{Dir}}$	0.73 ± 0.41	Fig. 8C
Indirect measurement by image analysis (*N* = 3)	Propellant acceleration rate (m/s^2^)	$a_{\text{Prop}}^{\mathrm{Img}}$	40.3 ± 3.7	-
	Whole propellant force calculated from $a_{\text{Prop}}^{\mathrm{Img}}$ (maximum value) (μN)	$F_{\text{Prop}}^{\mathrm{Img}}$	1,408 ± 161	-
	Rowing force of a middle leg calculated from $F_{\text{Prop}}^{\mathrm{Img}}$ (maximum value) (μN)	$F_{\mathrm{ML}}^{\text{Indir}}$	493 ± 56	Fig. 8A
	Maximum force arrival time (ms)	$t_{\text{maxF}}^{\text{Indir}}$	21 ± 4	Fig. 8B
	Angular velocity of leg rowing motion (deg/ms)	$\omega_{\mathrm{ML}}^{\text{Indir}}$	1.95 ± 0.24	Fig. 8C
	Maximum speed (mm/s)	$v_{\max}^{\text{Indir}}$	864 ± 153	-

**Table 3.** Summary of types of measurement methods and forces in water strider locomotion

Method		Direct force measurement		Indirect force measurement (calculated or estimated force)		
Type of force		Leg rowing force: $F_{\mathrm{ML}}^{\mathrm{Dir}}$	Leg rowing force calculated from propellant force	Leg rowing force: $F_{\mathrm{ML}}^{\text{Indir}}$	Leg rowing force (jumping)	Propellant force: $F_{\text{Prop}}^{\mathrm{Img}}$
Value	(μN)	955 (our study)	1,360–1,710 [25]300–400 [31]	493 (our study)500 [22]	-	1,408 (our study)13 [32]
	(μN/mm)	87 (our study)	-	45 (our study)80 [22]	144 [28]	-
	Nondimensional parameter	-	-	-	0.76–1.15 [26]	-

The second report of Goodwyn et al. [31] also calculated propellant force by using measured propellant force, and the calculated value was approximately 0.3 to 0.4 mN (300 to 400 μN) (Table 3). This value was smaller than in their first report [25], since the measurement system had been modified to cancel downward force. Their newly calculated rowing force was also smaller than our directly measured rowing force of a middle leg $F_{\mathrm{ML}}^{\mathrm{Dir}}$ despite the fact that they measured the force in all directions (not just the horizontal direction). Additionally, despite the larger weight of their water striders (50 to 56 mg) compared to this study (35.9 mg), our $F_{\mathrm{ML}}^{\mathrm{Dir}}$ was larger than their calculated force (propellant force linearly increases with water strider weight [25]). The difference might contribute to the transmission efficiency of propellant force. Our system could measure leg rowing force directly, and it could measure all the force generated by leg rowing motion. On the other hand, Goodwyn et al. [31] tethered water striders to a force transducer with a hair. When the water strider was rowing its legs, the legs slid on the water surface more than in the natural situation since the insect was tethered and it was prevented from being propelled. Additionally, propellant force was also decreased by friction force between the legs and water surface. Thus, propelling energy that was generated by leg rowing could be lost.

The force measured in this work $F_{\mathrm{ML}}^{\mathrm{Dir}}$ (955 μN) was also larger than the value (about 50 dynes = 500 μN) that was estimated by Hu et al. [22] (Table 3). This was despite our value only being for the horizontal direction force. This difference was caused by the difference of the species and weight of individuals. Hu et al. studied *Gerris remigis* with a weight of about 10 mg. On the other hand, our water striders were *A. paludum paludum* with a weight of 35.9 mg. As propellant force increases with water strider weight [25], we considered that rowing force was also increased with the weight. However, rowing force per unit length (80 dynes/cm = 80μN/mm [22]) was close to our measured force $F_{\mathrm{ML}}^{\mathrm{Dir}}$ (87 μN/mm; Table 3). There are 2 reasons for this. First, because *A. paludum paludum* is larger than *G. remigis*, the contact area between the legs and water surface increases more for the former. Second, because *A. paludum paludum* weighs more than *G. remigis*, the legs of *A. paludum paludum* sink deeper into the water and their contact area increases.

Feng et al. [2] measured the maximal supporting force of a single leg by the water surface. The maximum supporting force in the static condition (contact speed with a leg and water surface was 0.01 mm/s) was 152 dynes (1,520 μN), about 15 times the *G. remigis* weight. This value was larger than the rowing force $F_{\mathrm{ML}}^{\mathrm{Dir}}$, which was measured in this study (955 μN). Hu et al. [22] estimated maximal supporting force of a single leg as 140 dynes/cm (140 μN/mm) when the water strider moved on a water surface. This value was also larger than our measured force $F_{\mathrm{ML}}^{\mathrm{Dir}}$ (87 μN/mm; Table 3). Koh et al. [28] estimated supporting force in a more dynamic condition such as a jumping motion (escape motion) and found that the maximum supporting force of *A. paludum* was 144 mN/m (144 μN/mm) (if the leg force is larger than 144 mN/m, legs penetrate the water surface and jumping motion becomes unstable); this value was also larger than that of our study $F_{\mathrm{ML}}^{\mathrm{Dir}}$ (87 μN/mm; Table 3). This indicates that the water strider legs have a large margin for the legs not to penetrate the water surface. This fact suggests that rowing force is adjusted to an optimal value by natural selection or by individual learning. For confirmation, we derived the vertical direction force by image analysis and estimated the resultant middle leg rowing force (see Supplementary Report of Experiment including Figs. S6 to S8). The estimated resultant middle leg rowing force was 1.2 times greater than the maximum horizontal force. Therefore, we calculated the resultant middle leg rowing force as 1,146 μN (104 μN/mm). Because this value was smaller than the maximal supporting forces that were previously reported [2,22,28], we judged that the leg would not penetrate the water surface. In our experiments, sometimes the water strider’s opposite middle leg, which was not being used for the force measurement, penetrated the water surface. This might be due to the angle of the middle leg (the water strider might be connected to the force measurement system in a tilted condition) or changes of environmental conditions around the water surface (e.g., the wave collapsed because it could not be transmitted and the energy of the wave could not be diffused). This may indicate that the rowing force of water striders was set to the maximum value to get efficient locomotion.

Finally, we discuss indirect measurement by image analysis. The middle leg force and the angle of the middle leg changed simultaneously with the middle leg rowing motion (Fig. 7 and Fig. S4B to E). $F_{\text{Prop}}^{\mathrm{Img}}$ (1,408 ± 161 μN) was about 100 times larger than that reported by Steinmann et al. [32] (13 μN; Table 3). Steinmann et al. also calculated the whole propellant force from the acceleration rate, which was obtained by image analysis. The difference in the force might be due to the difference in water strider weight. Our water strider weight was about 100 times larger than that of Steinmann et al.’s study (our study, 35.9 mg; Steinmann et al.’s study, 0.35 mg). On the other hand, propellant acceleration rate $a_{\text{Prop}}^{\mathrm{Img}}$ was very close (our study, 40.3 m/s^2^; Steinmann et al.’s study, 39 m/s^2^). As a result, $F_{\text{Prop}}^{\mathrm{Img}}$ was also 100 times larger than that of Steinmann et al.’s study (our study, $F_{\text{Prop}}^{\mathrm{Img}}$ = 1,408 μN; Steinmann et al.’s study, 13 μN). This fact corresponded to the report that the propellant force was linearly increased with water strider weight [25]. This result implies that $a_{\text{Prop}}^{\mathrm{Img}}$ may be a constant value even if the water strider weight is different.

The direct force measurement that used the BAP could measure the middle leg rowing force alone because the indirect force measurement was affected by unnecessary forces that were not generated by the movement of middle leg rowing. Therefore, by comparing the middle leg rowing forces that were measured by the direct ($F_{\mathrm{ML}}^{\mathrm{Dir}}$) and indirect ($F_{\mathrm{ML}}^{\text{Indir}}$) force measurements, respectively, we could determine that about half the energy of $F_{\mathrm{ML}}^{\mathrm{Dir}}$ was lost. To summarize the energy losses to propellant force in the indirect force measurement, we offer an equation that allows explanation from the viewpoint of energy. All the types of propellant energy considered in the indirect force measurement (*E_p_*) are shown by Eq. 5.

$$E_{p}=E_{l}-\left(E_{c}+E_{v}+E_{d}+E_{b}+E_{a}\right)$$ (5)

*E_l_* is energy generated by the rowing legs for propelling. *E_l_* and *E_p_* related to the measured middle leg rowing force $F_{\mathrm{ML}}^{\mathrm{Dir}}$ and $F_{\mathrm{ML}}^{\text{Indir}}$, respectively. *E_c_*, *E_v_*, *E_d_*, *E_b_*, and *E_a_* are energies lost during propelling. *E_c_* is energy lost by the creation of capillary waves by the stroking legs (the capillary wave is used for purchase to get a foothold [23]). *E_v_* is energy lost by the deformation of the viscoelastic body (water). *E_d_* is energy lost by the drag between the legs and the water surface. *E_b_* is the energy lost by the bending of legs. *E_a_* is energy lost due to air resistance.

There are a few studies that have dealt with direct and indirect force measurements. By comparing different types of force measurements, we successfully clarified the differences of these forces measured directly and indirectly. Thus, we believe that our study can contribute to the fields of surface physics, biomechanics, and biology.

## Conclusions

In this study, we obtained the rowing force of a water strider’s middle legs by direct and indirect measurements. Because many previous studies have been based on estimated leg rowing force and lack some credibility, we tried to measure leg rowing force directly by a force transducer. To measure the water strider’s rowing leg force directly, a new measurement method and system were applied. Additionally, in order to decrease the effect of a meniscus, a BAP was developed. By using the measurement method and system and the BAP, the individual middle leg rowing force ($F_{\mathrm{ML}}^{\mathrm{Dir}}$: 955 ± 63 μN) was obtained by the direct force measurement. Then, to evaluate the directly measured leg force, we derived the leg rowing force ($F_{\mathrm{ML}}^{\text{Indir}}$: 493 ± 56 μN) by the indirect method (image analysis). $F_{\mathrm{ML}}^{\mathrm{Dir}}$ was about 2 times larger than $F_{\mathrm{ML}}^{\text{Indir}}$. We considered that half of the propellant energy was lost in the indirect force measurement by various other factors such as friction force between the legs and water surface, air resistance, strain of the legs, deformation of the water surface (viscoelasticity), and movement of the force application point.

## Ethics Approval

No ethics approval was required for this work.

## Data Availability

The data that support the findings of this study are available from the corresponding author upon reasonable request.

## Supplementary Materials

Supplementary 1 Supplementary Method Supplementary Analysis Supplementary Report of Experiment Table S1 and Figs. S1 to S8
