# Supplementary material for: Analysis of Rowing Force of the Water Strider Middle Leg by Direct Measurement Using a Bio-Appropriating Probe and by Indirect Measurement Using Image Analysis
Source: Cyborg Bionic Syst. 2023 Nov 17;4:0061. doi: 10.34133/cbsystems.0061 (PMC10655829; doi:10.34133/cbsystems.0061)
Supplement: Supplementary 1 — Supplementary Method Supplementary Analysis Supplementary Report of Experiment Table S1 and Figs. S1 to S8 [file cbsystems.0061.f1.docx]

**TITLE**

Analysis of rowing force of the water strider middle leg by direct measurement using a bio-appropriating probe and by indirect measurement using image analysis

**AUTHOR LIST**

Kaoru Uesugi^1,2,3^ (0000-0001-5266-3453), Hiroyuki Mayama^4^ (0000-0002-0496-5497), Keisuke Morishima^2,3^ (0000-0003-1146-3900)

**INSTITUTION IDENTIFICATION**

^1^Department of Mechanical Systems Engineering, Ibaraki University, 4-12-1 Nakanarusawa-cho, Hitachi, Ibaraki 316-8511, Japan

^2^Department of Mechanical Engineering, Osaka University, 2-1 Yamada-oka, Suita, Osaka 565-0871, Japan

^3^Global Center for Medical Engineering and Informatics, Osaka University, 2-1 Yamada-oka Suita, Osaka, 565-0871, Japan

^4^Department of Chemistry, Asahikawa Medical University, 2-1-1-1 Midorigaoka-Higashi, Asahikawa, Hokkaido 078-8510, Japan

**CORRESPONDING AUTHOR**

Kaoru Uesugi

E-mail: [kaoru.uesugi.biomech@vc.ibaraki.ac.jp](mailto:kaoru.uesugi.biomech@vc.ibaraki.ac.jp)

# Supplementary Table and Figures

**Table S1** Detailed data of the direct and indirect force measurements

| **Rowing force measured by the BAP [µN] (mean ± SD)** | | | |
| --- | --- | --- | --- |
| Direct force measurement $F_{ML}^{BAP}$ | Sample 1 (n = 3) | Sample 2 (n = 3) | Sample 3 (n = 10) |
|  | 2031 ± 482 | 2317 ± 65 | 2169 ± 364 |
| **Propellant acceleration rate [m/s^2^] and whole propellant force [µN] calculated from**  **the image analysis (mean ± SD)** | | | |
| Indirect force measurement $a_{Prop}^{Img}$ | Sample 4 (n = 9) | Sample 5 (n = 13) | Sample 6 (n = 15) |
|  | 41.1 ± 5.4 | 36.3 ± 8.1 | 43.5 ± 5.6 |
| Indirect force measurement $F_{Prop}^{Img}$ | Sample 4 (n = 9) | Sample 5 (n = 13) | Sample 6 (n = 15) |
|  | 1284.0 ± 168.9 | 1351.3 ± 300.4 | 1589.9 ± 205.9 |
| **Rowing force of middle leg [µN] (mean ± SD)** | | | |
| Direct force measurement $F_{ML}^{Dir}$ | Sample 1 (n = 3) | Sample 2 (n = 3) | Sample 3 (n = 10) |
|  | 883 ± 313 | 981 ± 45 | 1001 ± 165 |
| Indirect force measurement $F_{ML}^{Indir}$ | Sample 4 (n = 9) | Sample 5 (n = 13) | Sample 6 (n = 15) |
|  | 449 ± 59 | 473 ± 105 | 556 ± 72 |
| **Maximum force arrival time [ms] (mean ± SD)** | | | |
| Direct force measurement $t_{maxF}^{Dir}$ | Sample 1 (n = 3) | Sample 2 (n = 3) | Sample 3 (n = 10) |
|  | 207 ± 30 | 202 ± 165 | 72 ± 11 |
| Indirect force measurement $t_{maxF}^{Indir}$ | Sample 4 (n = 9) | Sample 5 (n = 13) | Sample 6 (n = 13) |
|  | 17.6 ± 3.3 | 24.6 ± 5.9 | 20.3 ± 4.5 |
| **Angular velocity of leg rowing motion [deg/ms] (mean ± SD)** | | | |
| Direct force measurement $\omega_{ML}^{Dir}$ | Sample 1 (n = 3) | Sample 2 (n = 3) | Sample 3 (n = 10) |
|  | 0.55 ± 0.22 | 0.44 ± 0.08 | 1.21 ± 0.28 |
| Indirect force measurement $\omega_{ML}^{Indir}$ | Sample 4 (n = 9) | Sample 5 (n = 11) | Sample 6 (n = 12) |
|  | 2.14 ± 0.47 | 1.68 ± 0.54 | 2.02 ± 0.43 |


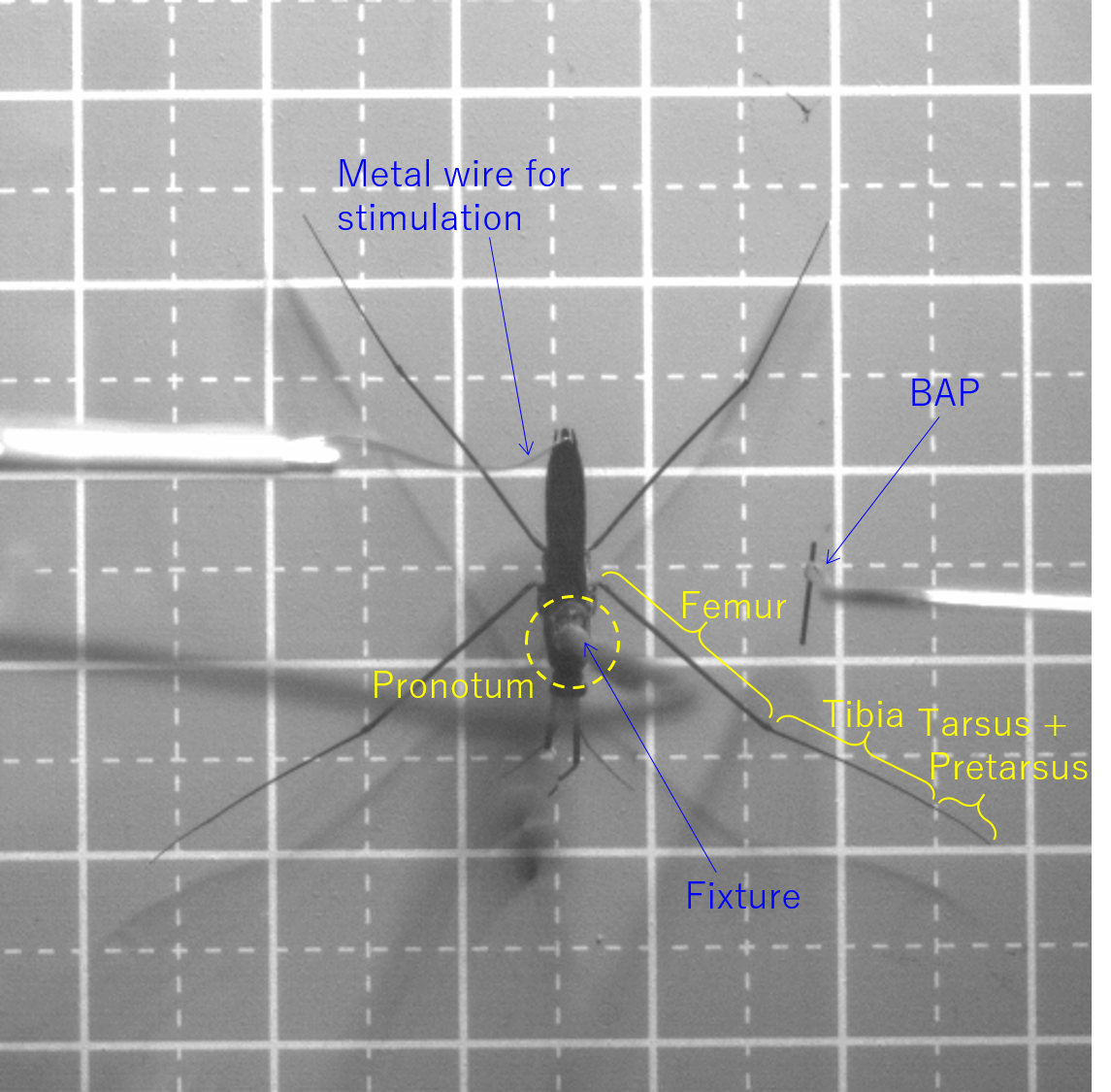


**Fig. S1** Regions of the water strider.

# Supplementary Method

**Attaching the fixture to the water strider body**

First, a water strider was placed in a covered 100 mm diameter polystyrene dish without water and the dish and insect were cooled in a refrigerator for 5-10 min to suppress the insect activity, but still keep it alive. Next, a fixture was temporarily adhered to the pronotum (Fig. S2) of the water strider using cyanoacrylate adhesive (ARON ALPHA Jelly-jyou #30523, Toagosei Co., Ltd., Tokyo) and the dish containing the insect with the fixture was put onto an ice pack for about 30 min to complete the adhesion between the fixture and the insect. Because there are few sensory hairs on the pronotum, the water strider does not sense the adhered fixture and does not act unnaturally. Additionally, the pronotum is mechanically tough and has a wide adhesion area. Finally, the adhered fixture was connected to the measurement system using the same cyanoacrylate adhesive.

The experiments were done at room temperature. The water strider with the adhered fixture was placed on the water surface in the pool of the measurement system (Fig. 3a, item 5) and the height between the water surface and mesosternum was 1.3–2.0 mm. The height between the water surface and mesosternum was measured before the leg force measurement for each water strider. The water strider freely floated on the water surface, and the height was measured with a specially made float having a carved scale (Fig. S2A). After this, the heights were confirmed and adjusted to the measured height by using the float (Fig. S2B). As the water strider was connected to the measurement system for a while at room temperature before measurements were made, the effect of the refrigerator storage and the ice pack application could be ignored.


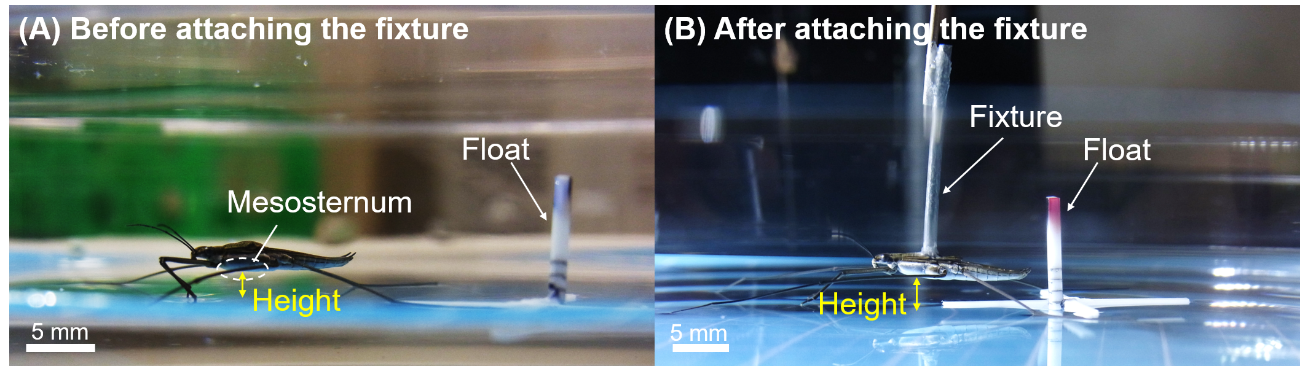


**Fig. S2** Photos showing measurement of height between the water surface and the mesosternum of a water strider. (A) The height was measured by the specially made float which had a carved scale. (B) After attaching the water strider to the fixture of the measurement system, the height was confirmed and adjusted to the measured height using the float.

# Supplementary Analysis

**Joint angles of the legs related to the single beam model**

We measured the size of the parts of the water strider using two types of image analysis software (Dipp-Motion V and ImageJ). The resolution of the high-speed images was 8.9376 pixels/mm.

We used the rowing force of the leg whose joint was not bent largely when the rowing leg touched the surface of the probe. Additionally, from the observation of the leg rowing of the water strider, we considered that no apparent active movement of the joints (femur-tibia and tibia-tarsus+pretarsus) occurred. During the time the leg temporarily touched the BAP until rowing was finished, small bending of its joints was observed (body-femur, 98.4 ± 8.2°; femur-tibia, 180.5 ± 3.1°; tibia-tarsus+pretarsus, 155.3 ± 30.7°) (Fig. S3). Therefore, for analysis of the moment, we hypothesized that the whole middle leg was a straight bar (two lengths were collinear) and we modeled the leg as a single beam with a constant moment. (The joint between the tibia-tarsus+pretarsus of one water strider might have been damaged slightly and the angle might be overestimated.)

Because there was a possibility that the legs bent unnaturally after touching the sensor probe, the joint angles of the legs and length were calculated at the instant the leg touched the probe.


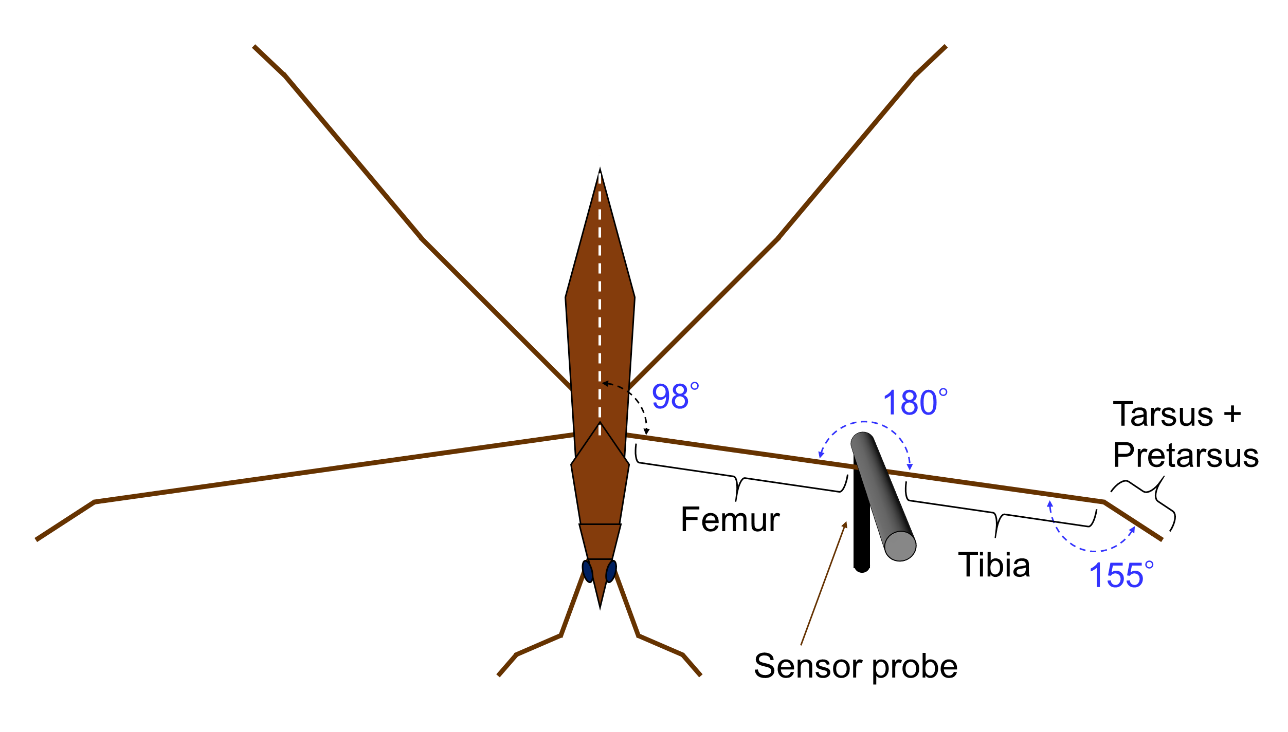


**Fig. S3** Schematic illustration showing the angle of each joint.

# Supplementary Results

(A)

(B)

(C)

(D)

(E)

**Fig. S4** Supplemental results of the direct and indirect measurements. (A) Typical results of the angular velocity of the water strider middle legs obtained by direct force measurement. (B) Typical image analysis results of propelled distance of the water strider. (C) Propellant velocity of water strider. (D) Propellant acceleration rate of water strider. (E) Angular velocity of the water strider middle legs.

# Supplementary Discussion


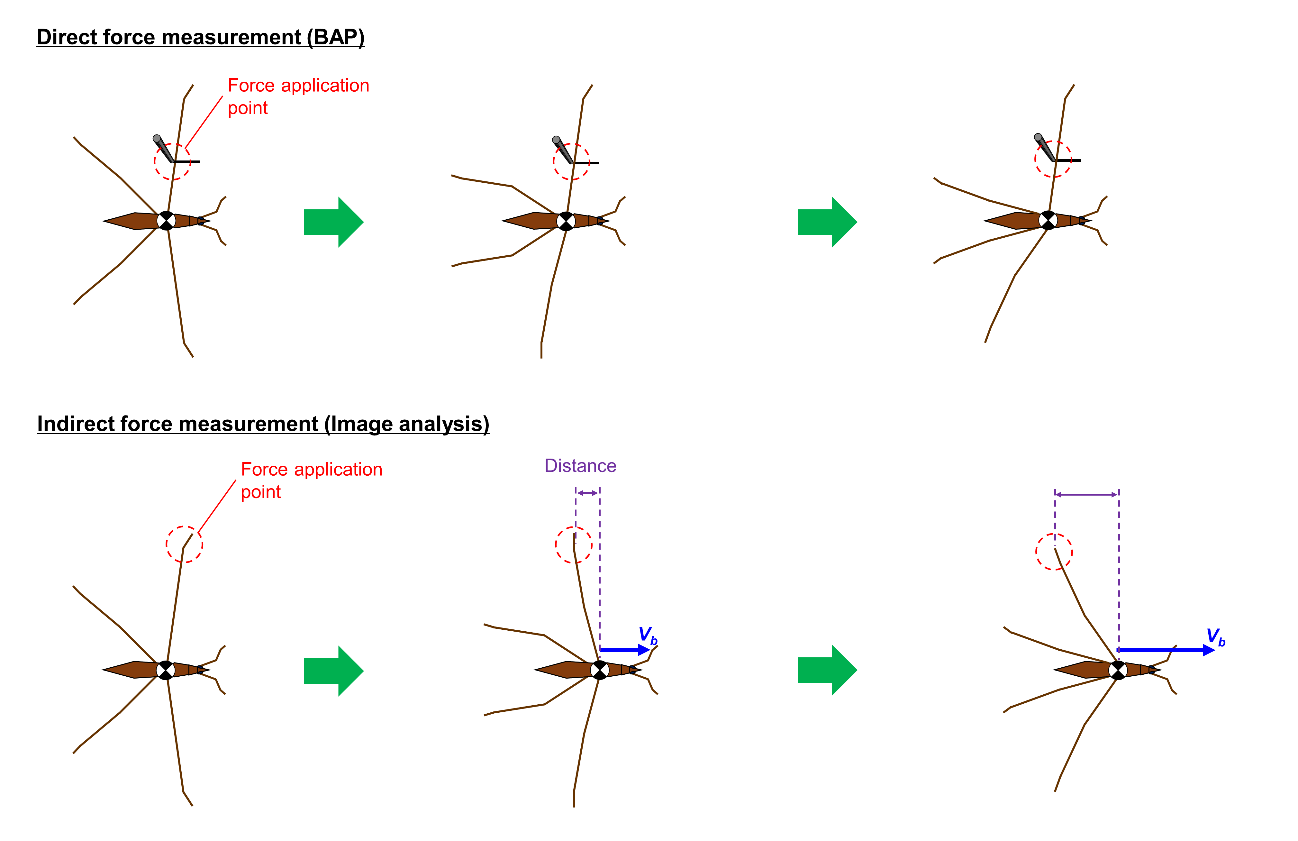


**Fig. S5** Relationship between the force application point and the water strider body. Because the force application point (capillary waves of the water surface made by the leg rowing motion) generally moved in the opposite direction to the propelled direction, the rowing speed of the middle leg tip could not catch up to the relative speed of the force application point. Consequently, the propellant force of the middle leg could not be transmitted to the water surface.

# Supplementary Report of Experiment

**Estimation of the vertical direction force**

We estimated the vertical directional force exerted by water striders while propelling themselves on a water surface. To carry out the estimation, we constructed an observation system that could record water striders from both the side and the top simultaneously using a high-speed camera (Fig. S6). The top-view images were obtained from the reflection of a mirror positioned at a 45-degree angle. We observed the propellant motions of a female water strider. The images were recorded at a rate of 500 fps 6 times for the same individual.


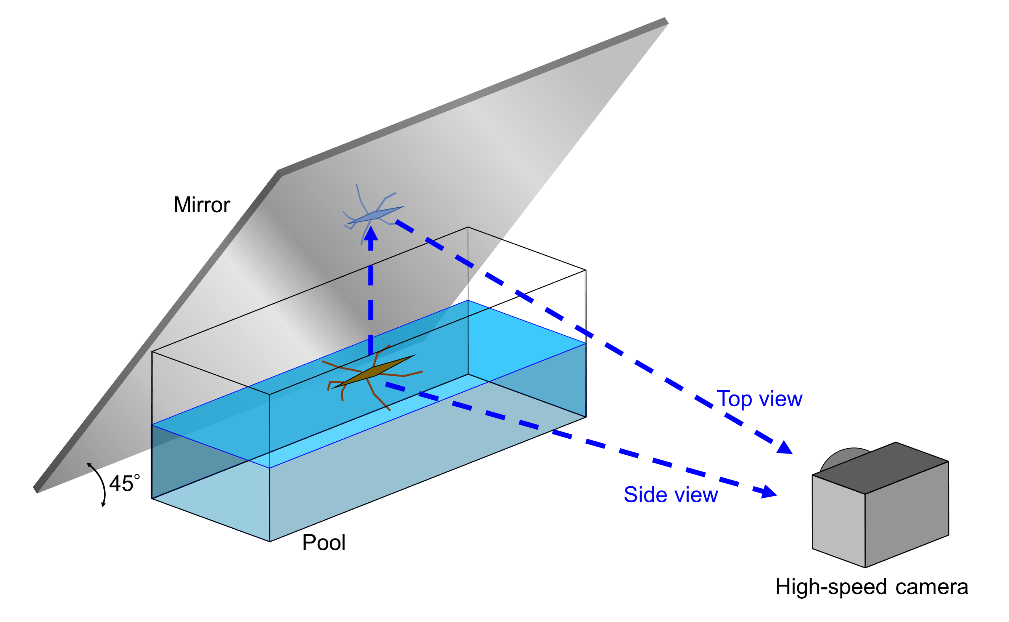


**Fig. S6** Schematic illustration of the experimental setup to observe the vertical and horizontal movements produced by propelling water striders. A high-speed camera was employed to capture both side-view and top-view images simultaneously. The top-view image was obtained by reflecting it onto a mirror set at a 45-degree angle.

Figure S7B,C shows the displacement, velocity, and acceleration rate of the water strider in both the horizontal and vertical directions. The propellant velocity (in the horizontal direction) and jumping velocity (in the vertical direction) of the water strider were determined by analyzing the displacement data (Fig. S7A) obtained using image analysis software (ImageJ). The acceleration rates in both horizontal and vertical directions were obtained by dividing the velocity differences by a short time interval (1/500 s).

The horizontal force (*F_H_*) and the vertical force (*F_V_*) were calculated using Eqs. S1 and S2, respectively.

$F_{H}={ma}_{H}$ (S1)

$F_{V}={ma}_{V}$ (S2)

Figure S8 is a schematic illustration explaining the resultant force generated during water strider propulsion. The variables *a_H_* and *a_V_* represent the acceleration rate of the horizontal and the vertical direction, respectively, during the rowing, and *m* denotes the mass of the water strider (33 mg). In the vertical direction, because the gravity force was loaded to the body of the water strider, the real jumping force *F_J_* of the water strider was calculated from Eq. S3.

$F_{J}=F_{V}+mg$ (S3)

Here, *g* represents the gravitational acceleration (9.8 m/s^2^). The maximum resultant propellant force *F_RP_* (Fig. S7D) was calculated using Eq. S4.

$F_{RP}=\sqrt{{F_{H}}^{2}+{F_{J}}^{2}}$ (S4)

Additionally, the resultant middle leg rowing force generated by a single middle leg ($F_{RPML}^{Img}$) was determined using Eq. S5 (see the “ANALYSIS” section).

$F_{RPML}^{Img}=0.7\times\frac{F_{RP}}{2}$ (S5)

The value of $F_{RPML}^{Img}$ (705 ± 91 µN) was 1.2 times greater than the maximum horizontal force $F_{HMax}^{Img}$ (575 ± 81 µN). We hypothesized that this relationship could also be applied to the middle leg rowing force $F_{ML}^{Dir}$ (955 µN) measured directly using the BAP, and we could estimate the resultant middle leg rowing force as $F_{RPML}^{Dir}$ (1146 µN (104 µN/mm)) from the $F_{ML}^{Dir}$ (Eq. S6).

$F_{RPML}^{Dir}=1.2\times F_{ML}^{Dir}$ (S6)


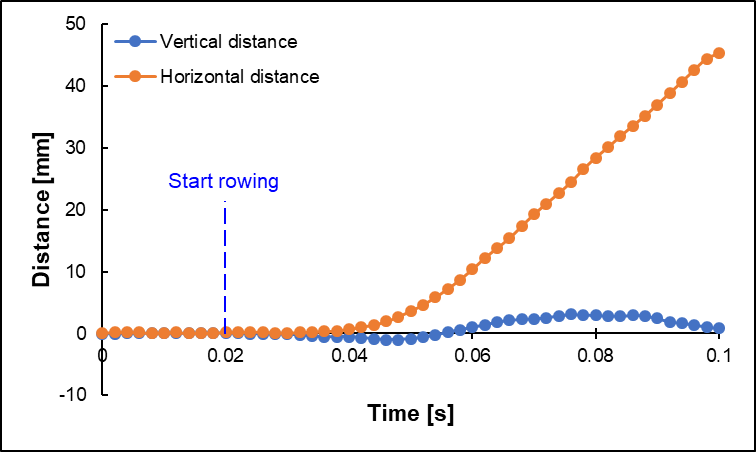

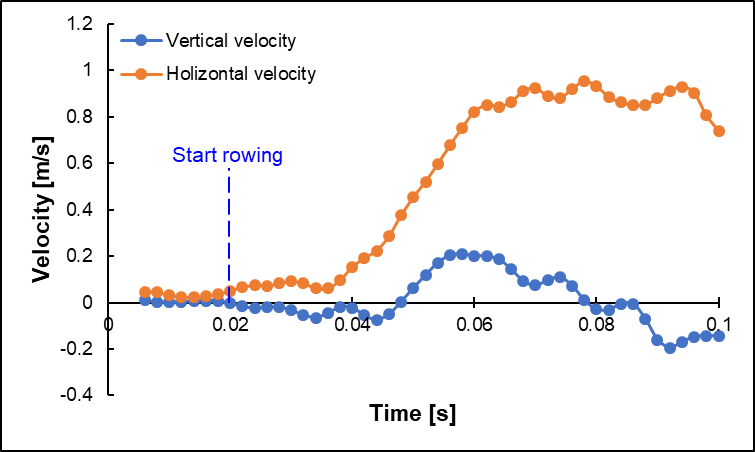


(A) (B)


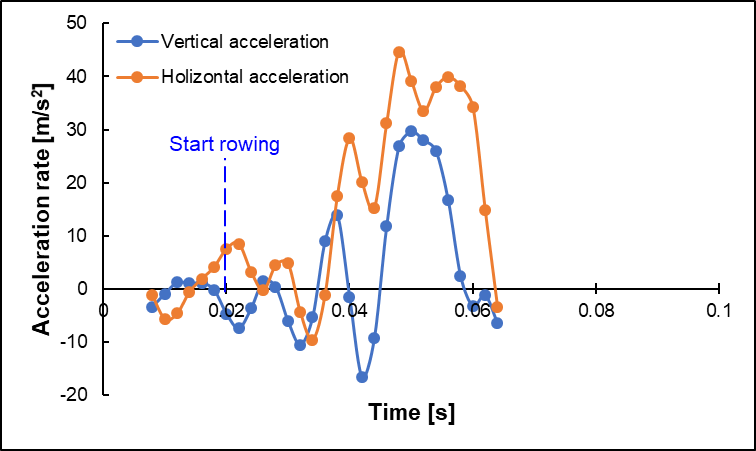

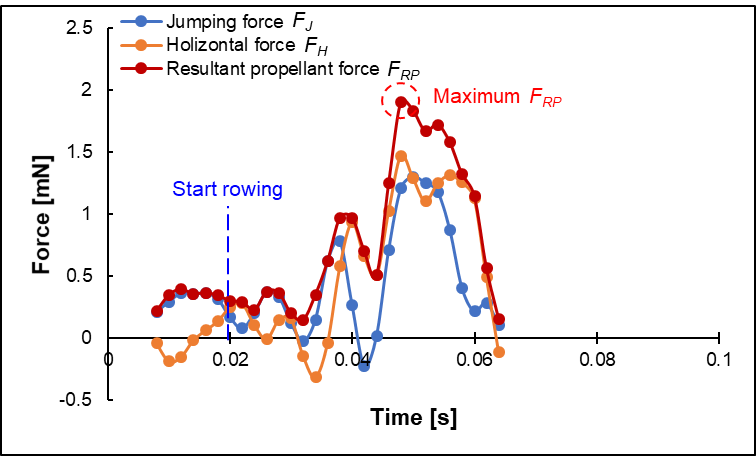


(C) (D)

**Fig. S7** Typical results showing the individual position (A), propellant velocity (B), acceleration rates in the horizontal and vertical directions (*a_H_*, *a_V_*) (C), and the resultant propellant force (*F_RP_*) (D).


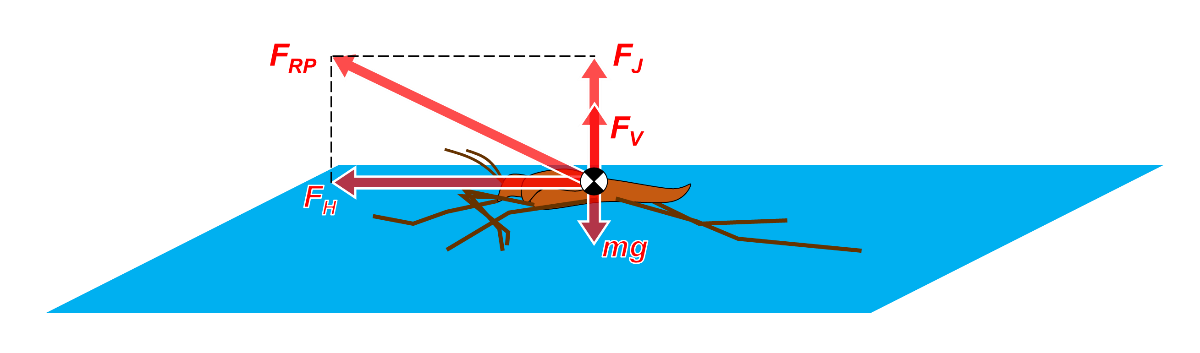


**Fig. S8** Schematic illustration explaining the resultant force generated during water strider propulsion. *F_H_* represents the propellant force in the horizontal direction, *F_J_* denotes the vertical direction force, *mg* corresponds to the gravitational force, *F_J_* is the jumping force obtained by summing *F_H_* and *mg*, and *F_RP_* is the resultant propellant force.

# Movie


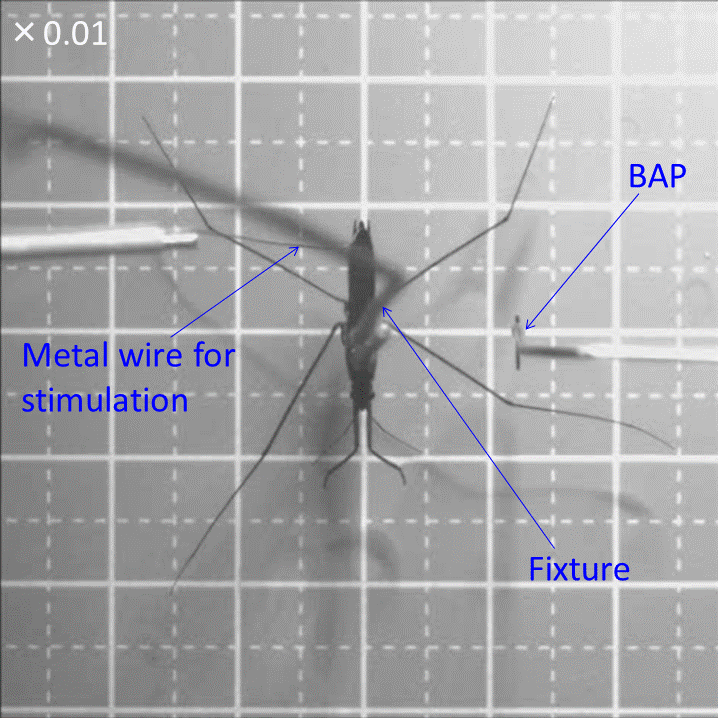


**Supplementary Movie 1** Multimedia view: State during the direct force measurement. The water strider attached to the fixture rowed its middle legs when the side of the abdomen or the caudal area was stimulated by touching the body area with a metal wire. Then, the moving middle leg was hooked by the BAP and its rowing force was measured. The movie is shown at 100 times the actual speed. The bold squares are 10 mm on a side.
